# Evidence for a Hydrogenosomal-Type Anaerobic ATP Generation Pathway in *Acanthamoeba castellanii*

**DOI:** 10.1371/journal.pone.0069532

**Published:** 2013-09-27

**Authors:** Michelle M. Leger, Ryan M. R. Gawryluk, Michael W. Gray, Andrew J. Roger

**Affiliations:** Department of Biochemistry and Molecular Biology, Centre for Comparative Genomics and Evolutionary Bioinformatics, Dalhousie University, Halifax, Nova Scotia, Canada; University of South Alabama, United States of America

## Abstract

Diverse, distantly-related eukaryotic lineages have adapted to low-oxygen environments, and possess mitochondrion-related organelles that have lost the capacity to generate adenosine triphosphate (ATP) through oxidative phosphorylation. A subset of these organelles, hydrogenosomes, has acquired a set of characteristic ATP generation enzymes commonly found in anaerobic bacteria. The recipient of these enzymes could not have survived prior to their acquisition had it not still possessed the electron transport chain present in the ancestral mitochondrion. In the divergence of modern hydrogenosomes from mitochondria, a transitional organelle must therefore have existed that possessed both an electron transport chain and an anaerobic ATP generation pathway. Here, we report a modern analog of this organelle in the habitually aerobic opportunistic pathogen, *Acanthamoeba castellanii.* This organism possesses a complete set of enzymes comprising a hydrogenosome-like ATP generation pathway, each of which is predicted to be targeted to mitochondria. We have experimentally confirmed the mitochondrial localizations of key components of this pathway using tandem mass spectrometry. This evidence is the first supported by localization and proteome data of a mitochondrion possessing both an electron transport chain and hydrogenosome-like energy metabolism enzymes. Our work provides insight into the first steps that might have occurred in the course of the emergence of modern hydrogenosomes.

## Introduction

The capacity to produce adenosine triphosphate (ATP) under low oxygen conditions is found throughout the eukaryote tree, in diverse, distantly-related organisms. Of the lineages of this type that have been studied, most are anaerobic or microaerobic, and possess mitochondrion-related organelles (MROs), which, although derived from mitochondria, have lost the capacity to generate ATP through oxidative phosphorylation (reviewed in [1]). Some of these organelles, known as hydrogenosomes, have adopted a new function in anaerobic ATP generation by acquiring a set of characteristic enzymes that are commonly found in anaerobic bacteria [2]–[4]. In other anaerobic/microaerobic eukaryotes with more highly reduced MROs, such as *Giardia intestinalis* and *Entamoeba histolytica*, homologous enzymes are localized in the cytosol, and the MROs of these organisms are not involved in ATP generation [5], [6]. MROs have long been classified according to their role in energy metabolism, and a recent review [7] retains the categories of mitochondria (class 1 under the authors' classification system), hydrogenosomes (class 4) and mitosomes (class 5), while proposing new classes to formally accommodate the more diverse range of MROs now known: anaerobically functioning mitochondria that do not produce hydrogen (class 2) and mitochondria that both possess an electron transport chain and produce hydrogen (class 3).

The best-characterized hydrogenosomes are those of the obligately parasitic parabasalid *Trichomonas vaginalis*. In mitochondria, the first step of pyruvate catabolism involves the decarboxylation of pyruvate by the enzyme complex pyruvate dehydrogenase (PDH, EC 1.2.1.51), which produces acetyl-CoA and CO_2_ with the concomitant reduction of NAD^+^ to NADH. In the hydrogenosomes of *T. vaginalis*, pyruvate decarboxylation is instead carried out by pyruvate:ferredoxin oxidoreductase (PFO, EC 1.2.7.1) [8], [9]. In other eukaryotes such as *Cryptosporidium parvum*, *Neocallimastix frontalis* or *Mastigamoeba balamuthi*, a similar function is performed by a pyruvate:NADP oxidoreductase (PNO, EC 1.2.1.51) [10], [11] or pyruvate:formate lyase (PFL, EC 2.3.1.54) [12], [13]. Electrons produced during pyruvate decarboxylation by PFO are transferred first to a [2Fe-2S] ferredoxin, and then to [FeFe]-hydrogenase (EC 1.12.1.4), which reduces protons, producing molecular hydrogen. Three maturases, HydE, HydF and HydG, are involved in the assembly and insertion of the catalytic iron-sulfur clusters in bacterial [FeFe]-hydrogenases, and homologs of these enzymes have been reported in some, though not all, eukaryotes possessing this enzyme [14]–[16]. Coenzyme A is transferred from the resulting acetyl-CoA to succinate by an acetate:succinate CoA transferase (ASCT, EC 2.8.3.8) [17], generating acetate and succinyl-CoA. The subsequent regeneration of succinate, catalyzed by the tricarboxylic acid (TCA) cycle enzyme succinate thiokinase (STK, also referred to as succinyl-CoA synthetase, EC 6.2.1.4), generates ATP through substrate-level phosphorylation [4]. ASCTs found in eukaryotes have been classified into three subfamilies [17]. Enzymes of subfamily 1A, found in the mitochondria of trypanosomatids [18] and in the hydrogenosomes of rumen ciliates [19], are homologous to the succinyl-CoA:3-oxoacid CoA transferases (SCOT) that are involved in ketone body degradation in metazoan mitochondria. Members of subfamily 1B have been reported in the mitochondria of acetate-producing metazoans such as *Fasciola hepatica* [20] and *Artemia franciscana* [21]. Based on *in silico* predictions, they are also believed to be present in *Blastocystis* sp. [17] and in the mitochondria of *Naegleria gruberi* [22]. Enzymes of subfamily 1C have been found in the hydrogenosomes of *T. vaginalis* [23], *Blastocystis* sp. [24], [25], and the chytrid fungus Neocallimastix [17], [26].

Until recently, the only aerobic eukaryotes known to possess both [FeFe]-hydrogenase and PFO were green algae such as *Chlamydomonas reinhardtii* and *Scenedesmus* spp. In these organisms, [FeFe]-hydrogenase and PFO are expressed upon exposure to anoxic conditions, and localize to the chloroplast, where they function in both anaerobic energy production and anaerobic photosynthesis [27]–[29]. In 2010, genes encoding an [FeFe]-hydrogenase and the three [FeFe]-hydrogenase maturases were identified in the genome of *Naegleria gruberi*, an aerobic heterolobosean; *in silico* predictions suggested that these enzymes might be mitochondrially targeted [22]. No PFO homologs have been found in the genome of this organism.

Previous studies have attempted to clarify the origin of these enzymes in eukaryotes; these efforts have generally been hampered by the small number of eukaryotic sequences available, and by low resolution in all parts of the tree. Phylogenetic analyses of [FeFe]-hydrogenase sequences have consistently recovered more than one eukaryotic clade, suggesting at least two origins of these enzymes in eukaryotes [16], [30]–[33]. A specific relationship between eukaryotic [FeFe]-hydrogenases and their homologs in α-proteobacteria has been rejected in topology tests, providing evidence against a mitochondrial endosymbiotic origin of [FeFe]-hydrogenases in extant eukaryotes [16]. Analyses of [FeFe]-hydrogenase maturases recovered robust eukaryotic clades in all cases; however, the internal relationships within these clades were poorly supported and their closest prokaryotic homologs were, in no case, α-proteobacterial. Similar results were obtained by phylogenetic analyses of PFO [16], [34]–[36]. A neighbor-net analysis of ASCT1B and ASCT1C sequences [23] recovered eukaryote monophyly for both enzymes, consisting of metazoa in the case of ASCT1B, and fungi and *T. vaginalis* in the case of ASCT1C; at that time these taxa were the only eukaryotes known to possess ASCTs. Again, no clear α-proteobacterial affinity for eukaryotic groups was recovered, and thus there is no clear connection to mitochondrial origins. These observations suggest that lateral gene transfer has played a role in the appearance of these enzymes within eukaryotes; however the number of events involved, and the precise nature of the donor and recipient lineages, remain unclear.

*Acanthamoeba castellanii* is a free-living soil amoeba, found in a diverse range of marine, freshwater, soil and human-related environments. As an opportunistic pathogen, it is responsible for amoebic keratitis and granulomatous amoebic encephalitis in humans [37], and under free-living conditions, it grazes on bacterial biofilms [38]. Thus it is likely that *A. castellanii* routinely encounters anaerobic or microaerobic conditions. Furthermore, while this amoebozoan has been reported to encyst rapidly when exposed to degassing with N_2_ [39], it is now known to respond well to low-oxygen conditions, replicating faster under these conditions than under aerobic ones [38].

In 2010, Hug *et al.* reported partial sequences of a few enzymes associated with anaerobic ATP generation in publicly available expressed sequence tag (EST) data from *A. castellanii* [16]. Here, we report the existence a complete ‘hydrogenosomal’ type anaerobic ATP generation pathway, describe the genomic and transcript sequences of all enzymes involved, and show that they all possess classical mitochondrial targeting peptides. We show, by tandem mass spectrometry, that three of these enzymes – PFO, ASCT1B, and the [FeFe]-hydrogenase maturase HydF – are found in the mitochondria of aerobically grown cells. Our findings confirm the presence of a complete hydrogenosome-like ATP generation pathway in *A. castellanii* and strongly suggest that the enzymes are present within the mitochondria of this organism. Our results raise the tantalizing possibility that the mitochondrion of *A. castellanii* is able to act as an organelle with two metabolic modes, producing energy either aerobically, via classical oxidative phosphorylation, or anaerobically, via a hydrogenosomal-type pathway, according to the environmental conditions that the amoeba encounters.

## Materials and Methods

### EST assembly

454 ESTs available through the Baylor College of Medicine Human Genome Sequencing Center (http://www.hgsc.bcm.tmc.edu/ftp-archive/AcastellaniNeff/ESTs/) were assembled using CAP3 [40] and Mira [41], [42].

### Database searching

Partial EST sequences of [FeFe]-hydrogenase, PFO, HydE and HydG previously identified by Hug *et al.* [16] were retrieved by performing Basic Local Alignment Search Tool (BLAST [43]) searches against the publicly available *A. castellanii* expressed sequence tag (EST) library at TBestDB [44]. Genomic sequence data were obtained by performing subsequent BLASTn searches against scaffolds assembled by the Baylor College of Medicine Human Genome Sequencing Center (http://www.ncbi.nlm.nih.gov/bioproject/PRJNA20303; see Table S1 for accession numbers), using the *A. castellanii* ESTs as queries. As no ESTs encoding HydF or an ASCT had been identified, we used a *Clostridium kluyveri* HydF protein sequence (EDK34342) and *Trypanosoma brucei*, *Fasciola hepatica* and *Trichomonas vaginalis* ASCT sequences (EAN79240, ACF06126 and XP_001330176, respectively) as heterologous query sequences for tBLASTn searches. Subsequently, full-length cDNA sequences were obtained by performing BLASTn searches against the 454 EST data, using previously identified EST or genomic sequences as queries. The identities of hits were verified by performing BLASTx searches against GenBank. Start codons and intron positions were verified manually. Predicted mitochondrial targeting peptides were identified using TargetP [45]–[47].

### Cell culture

Cells were maintained at room temperature, but otherwise as described in [48].

### Tandem mass spectrometry

Mitochondria were isolated from aerobically grown cells, purified on sucrose gradients, and subfractionated as described in [49]. A whole mitochondrial fraction (SWM), a soluble protein-enriched fraction (SPE), and a mitochondrial membrane protein-enriched fraction (MPE) were separated on sodium dodecyl sulfate-polyacrylamide gel electrophoresis (SDS-PAGE) gels; the resulting lanes were excised in approximately equally sized bands and digested, as described in [49]. These fractions were separated by reverse-phase high-performance liquid chromatography (HPLC) and subjected to tandem mass spectrometry (MS/MS) as described [49]. An additional whole mitochondrial fraction that had not undergone SDS-PAGE was digested in solution (WM) and separated into fractions using strong cation exchange liquid chromatography; these fractions were resolved by reversed-phase HPLC and subjected to MS/MS. Data were acquired and analyzed against the genomic and 454 EST data using Mascot [50], as described in [51]. Of the 143 nuDNA-encoded proteins identified by MS/MS in [49], representing respiratory chain proteins (88 proteins) and non-respiratory chain proteins that contaminated preparations of respiratory complexes (55 proteins), none were known non-mitochondrial contaminants and none were confidently predicted to possess sorting signals that direct proteins to other cellular compartments, such as the endoplasmic reticulum or peroxisomes; accordingly, this protocol was deemed to have produced sufficiently pure mitochondria samples. In order to confirm that the samples used in this particular experiment were highly enriched in mitochondria, we compared the gel electrophoretic profile of the rRNA species recovered from the purified mitochondria to that of total cellular RNA, as a proxy for protein profiles (Figure S1). The RNA profiles were distinct; in particular, we were unable to detect cytosolic LSU or SSU species in our purified mitochondrial samples, indicating a high degree of mitochondrial enrichment in these samples.

### Phylogenetic analyses

For each protein, NCBI databases of proteins and EST sequences were searched using BLASTp or tBLASTx respectively, and added to preexisting datasets used in [16] where applicable. Preliminary alignments were made using MUSCLE [52], [53], FSA [54] or MAFFT-L-INS-I [55]–[57] and trimmed using BMGE [58] or a script written by Dr. Daniel Gaston. Preliminary trees were made using FastTree [59], or RAxML (version 7.2.6 [60], using the Le and Gascuel [LG] model of amino acid substitution rates [61] with empirical amino acid frequencies and the gamma model of rate heterogeneity [PROTGAMMALGF]. Based on these initial analyses, long-branching taxa and paralogs were eliminated. In particular, a long-branching clade comprising both eukaryotic and bacterial sequences was identified in [FeFe]-hydrogenase analyses. Final analyses were performed both with and without these sequences. Initially, ASCT1C sequences were analyzed together with distantly homologous ASCT1B sequences, and distantly homologous HydE and HydF sequences were analyzed together; this was done to better identify paralogs in the face of widespread misannotation in GenBank.

Final alignments were made using MAFFT-L-INS-I, verified manually, and trimmed using BMGE. Independent maximum likelihood (ML) trees (200) and 1000 bootstrap replicates were generated in RAxML [PROTGAMMALGF] model, and bootstrap values were mapped onto the best-scoring tree. Bayesian inference posterior probabilities were calculated using PhyloBayes [62] under the [catfix C20] model of evolution [63].

### Topology tests

We tested support for various grouping topologies using the approximately unbiased (AU) test in CONSEL [64]. For each hypothesis tested, five ML trees were generated for a given constraint tree, using the PROTGAMMALGF model and the –g option in RAxML. Subsequent CONSEL analyses used the 1000 bootstrap trees initially produced to generate p-values for the best trees generated from the constrained RAxML analyses.

## Results

### The *A. castellanii* genome encodes a complete anaerobic ATP generation pathway similar to that found in *T. vaginalis* hydrogenosomes

Hug *et al.* [16] had identified partial sequences of [FeFe]-hydrogenase, PFO, HydE and HydG in the *A. castellanii* transcriptome (Table S1). We have identified corresponding genomic sequences for all four genes. Searches using a *Clostridium* HydF sequence and ASCT sequences from *Trypanosoma brucei*, *Fasciola hepatica* and *Trichomonas vaginalis* as queries yielded a HydF homolog and two possible candidates for an ASCT, homologous to the *T. brucei* (subfamily 1A) and the *F. hepatica* (subfamily 1B) enzymes. The subfamily 1A enzyme of *T. brucei* is homologous to succinyl-CoA:3-ketoacid-CoA transferase (SCOT), an enzyme that is widespread in mammalian and fungal mitochondria, and that catalyzes the transfer of CoA from succinyl-CoA to a 3-oxoacid. The top BLASTp hits for this candidate were SCOT homologs from *Polysphondylium pallidum* and *Dictyostelium discoideum*, two other, ‘cellular slime mold’, amoebozoans that do not appear to possess anaerobic ATP generation enzymes. In contrast, the *F. hepatica*-type enzyme has been described in platyhelminths and arthropods, and is homologous to bacterial 4-hydroxybutyrate CoA-transferases; we were unable to find homologs of this enzyme in the *Polysphondylium pallidum* or *Dictyostelium* genome sequences available through dictyBase [65]. Accordingly, we concluded that the *F. hepatica* enzyme hit was the more likely candidate to function in a pathway with the other anaerobic enzymes we had discovered. The *A. castellanii* genome encodes a single adrenodoxin-like [2Fe-2S] ferredoxin, homologous to eukaryotic mitochondrial ferredoxins, which may function as the electron mediator from PFO to [FeFe]-hydrogenase; and STK, which may perform dual functions in the TCA cycle and in anaerobic ATP generation.

**Figure 1:**
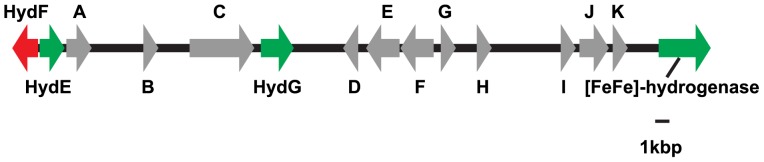
Map of the genomic segment encoding [FeFe]-hydrogenase and its associated maturases. The direction of the arrows indicates the transcriptional orientation of the genes. Green, anaerobic metabolism enzyme-encoding gene located on the forward strand. Red, anaerobic metabolism enzyme-encoding gene located on the reverse strand. Gray, predicted gene encoding a product not evidently involved in anaerobic metabolism. Predicted genes annotated according to top BLAST hits in dictyBase: A. Similar to interferon-related protein PC4-like. B. Region with similarity to *Dictyostelium* hypothetical protein, possibly truncated at the 5′ end. C. Similar to importin beta 4. D. Low similarity to *Dictyostelium* vasodilator-stimulated phosphoprotein. E. Similar to RNA-binding region RNP-1 domain-containing protein. F. Similar to molybdenum cofactor synthesis protein 1. G. Similar to molybdenum cofactor synthesis protein 2. H. Region with similarity to sequences annotated as hypothetical protein in GenBank, but without tBLASTx hits in dictyBase. I. Similar to PHD Zn finger-containing protein. J. Low similarity to *Dictyostelium* hypothetical protein. K. Region without significant hits in either GenBank or dictyBase, but which corresponds to EST sequence and apparently contains an intron.

Full-length EST sequences for each of these enzymes were retrieved from 454 pyrosequencing data, confirming the locations of spliceosomal introns in the genomic sequences. All of the genomic sequences contained canonical 5′GT-AG3′ spliceosomal introns, refuting the possibility that the genes we identified originated from bacterial contamination of the transcriptomic and genomic data (see Table S1 for the list of accession numbers). Interestingly, the genes encoding [FeFe]-hydrogenase and all three maturases are encoded within a single ∼50-kb stretch of the genome (Figure 1). Within this region, 11 other predicted genes were found that had homologs returned by BLAST searches. With the exception of two genes encoding hypothetical proteins, all of the predicted genes had significant tBLASTx hits (with an E-value≤10^−3^) to one of more of the amoebozoan genomes available through dictyBase. None of these additional genes has any obvious function in anaerobic respiration.

**Figure 2:**
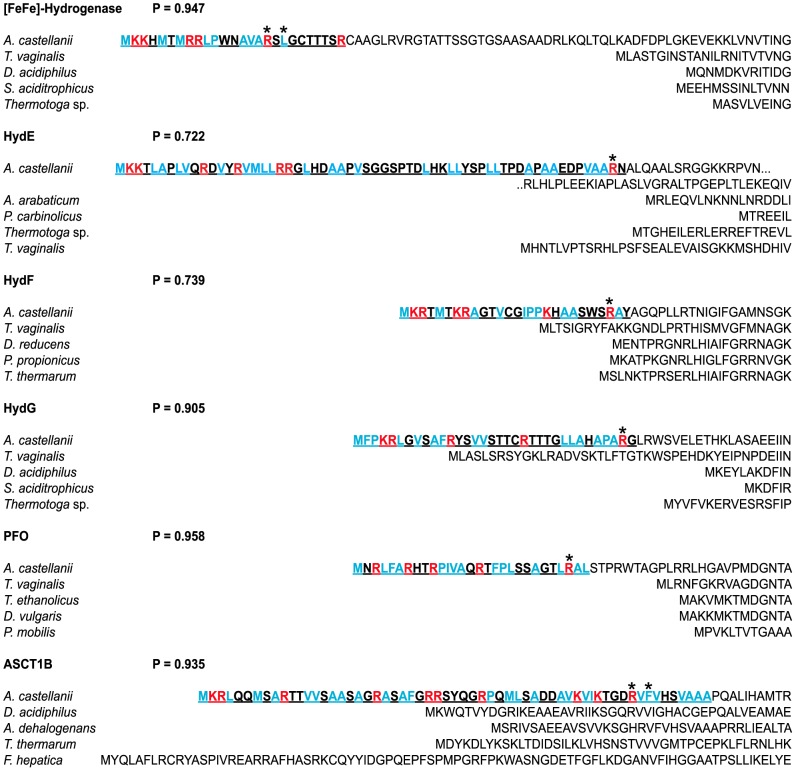
N-termini of anaerobic energy generation enzymes in *A. castellanii*, showing putative mitochondrial targeting peptides (mtTPs). Putative mtTPs were predicted by TargetP, and are shown underlined and bold. N-termini of bacterial and eukaryotic homologues are shown for comparison; bacterial homologues lack targeting peptides. Positively charged residues in the predicted mtTPs are shown in red; hydrophobic residues are shown in blue. Arginine residues at positions −2, −3 or −10, and hydrophobic residues at position −8, believed to be important in determining the cleavage site [66], are marked with an asterisk. P, TargetP mitochondrial targeting probability.

TargetP predicted high probabilities of mitochondrial localization, and identified putative targeting peptide cleavage sites, for all of the anaerobic ATP generation enzymes (Figure 2). The predicted mitochondrial targeting peptides (mtTPs) are rich in hydrophobic and positively charged amino acids, consistent with the amphipathic helix structure that mitochondrial targeting peptides are known to adopt [66]. The mtTPs have arginine residues at positions −2, −3 or −10 relative to the cleavage site, as well as positively charged residues at position −8 in the latter case. Such residues are believed to be important in determining the site of targeting peptide cleavage [66]. The composition of the predicted targeting peptides is consistent with those predicted by TargetP for other nucleus-encoded proteins known to be mitochondrially targeted in *A. castellanii*, such as mitochondrial malate dehydrogenase, dihydrolipoamide dehydrogenase and isocitrate dehydrogenase (data not shown).

### PFO, ASCT1B and the [FeFe]-hydrogenase maturase HydF are present in the mitochondrial proteome

**Figure 3:**
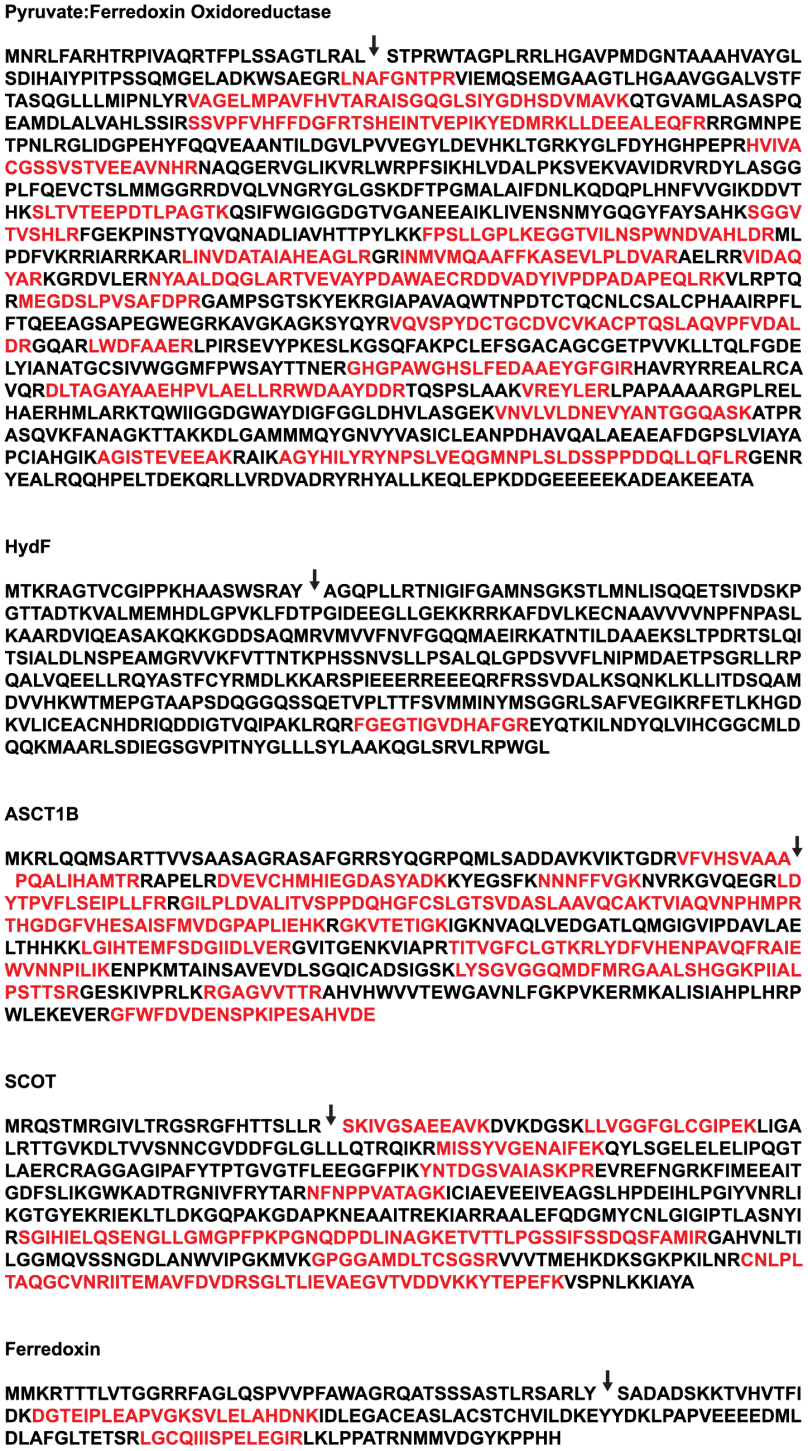
Peptides identified in tandem mass spectrometry experiments mapped to anaerobic ATP generation enzyme sequences. Peptides identified in mitochondrial fractions are shown in red. mtTP cleavage sites predicted by TargetP are shown with arrows.

Peptides diagnostic of PFO, ASCT1B and HydF were detected in the mitochondrial protein fractions by tandem mass spectrometry (Table 2, Figure 3).

**Table 2:** Anaerobic ATP generation enzymes identified in tandem mass spectrometry experiments.

	Unique peptides	Ion score^1^	Fractions
[FeFe]-hydrogenase	N/A	N/A	None
HydE	N/A	N/A	None
HydG	N/A	N/A	None
HydF	1	59	WM
PFO	30	1457	WM, SWM, SPE
ASCT1B	17	1900	WM, SWM, SPE
SCOT	14	1631	WM, SWM, SPE
Ferredoxin	3	141	SPE

1 P<0.05 for ion score≥3.

Thirty unique PFO-specific peptides were identified in the WM, SWM and SPE fractions, with a high ion score (1457), evidence that PFO is present at a relatively high abundance in *A. castellanii* mitochondria even under aerobic conditions. No PFO-specific peptides were identified in the MPE fraction; these findings are consistent with PFO in *A. castellanii* being a soluble matrix protein, in contrast to that of *T. vaginalis*, which is bound to the hydrogenosomal membrane [67]. ASCT1B was similarly well represented in the mitochondrial proteome (ion score: 1900, 17 unique peptides).

A single peptide from the [FeFe]-hydrogenase maturase HydF was identified, as were three peptides from ferredoxin. No peptides corresponding to [FeFe]-hydrogenase, or to the two other [FeFe]-hydrogenase maturases, were recovered.

In addition, we performed immunogold labeling experiments on *A. castellanii* cells that had been exposed to anaerobic conditions for 6 or 24 hr, using an antibody raised against *A. castellanii* [FeFe]-hydrogenase (Methods S1). Antibody staining in these cells was elevated in mitochondria (approx. 2.9-fold higher than in the cytosol, and approx. 1.7-fold higher than in the nucleus), consistent with the presence of a predicted mitochondrial targeting peptide for [FeFe]-hydrogenase (Figures S2, S3, S4), although the high levels of antibody required and the degree of cross-reaction in other cellular locations suggest that optimal expression conditions remain to be established for [FeFe]-hydrogenase in *A. castellanii*.

### Evolutionary histories of anaerobic ATP generation enzymes

**Figure 4:**
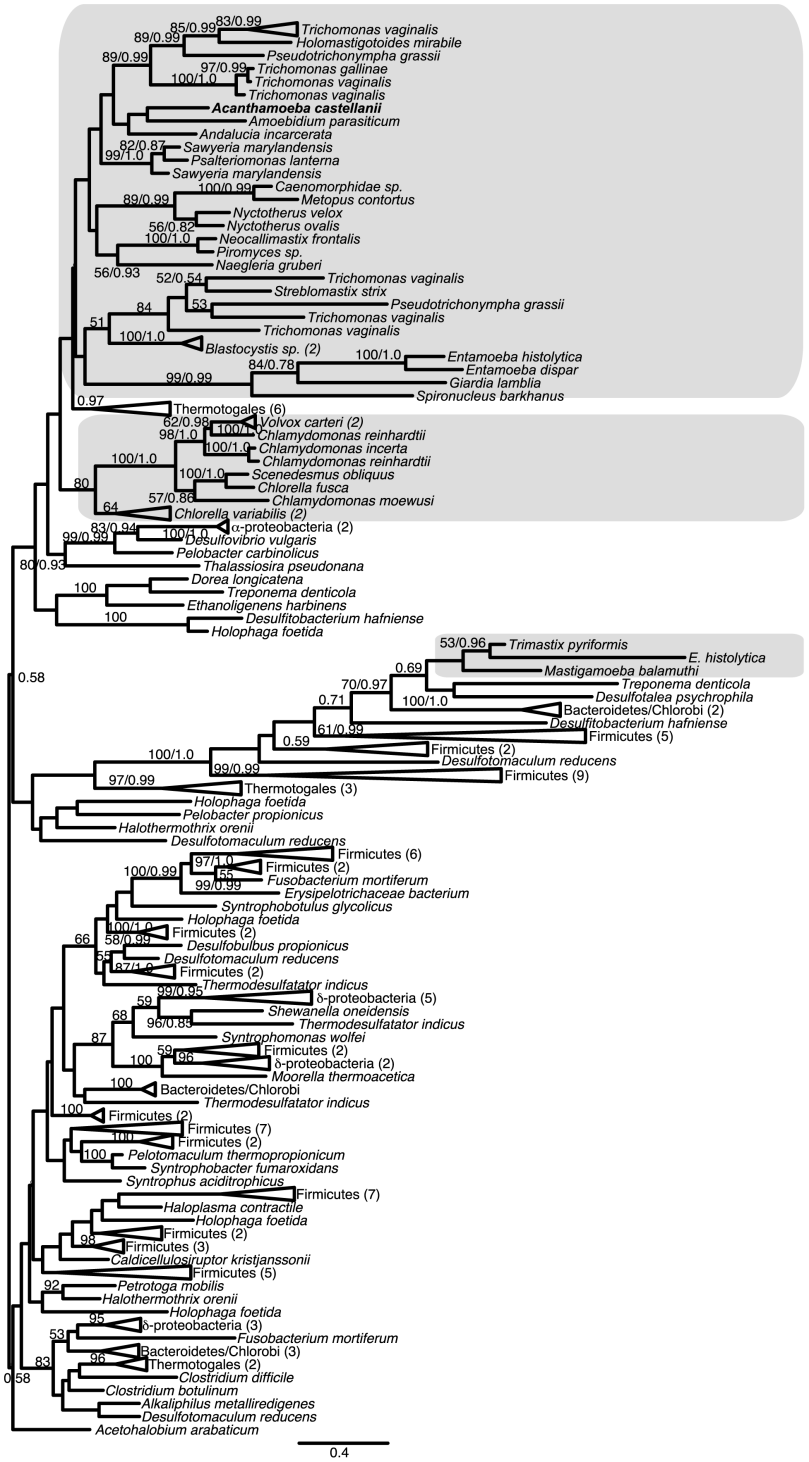
Phylogeny of [FeFe]-hydrogenase in eukaryotes and bacteria. The topology shown is the maximum likelihood (ML) tree generated by RAxML analyses; 279 sites were examined across 175 taxa. Bootstrap support values ≥50% and Bayesian posterior probabilities ≥0.5 are shown. Eukaryotes are shaded gray.

Previous analyses of [FeFe]-hydrogenase phylogenies have failed to recover eukaryotes as a monophyletic clade [16], [30], [68], [69]. Our results (Figure 4), which include sequences from a larger number of eukaryotic taxa than were previously available, are consistent with these findings, in that we recover at least three distinct eukaryotic clades. While our trees suffer from the same poor resolution (i.e. low bootstrap support for many branches) that has been reported in previous analyses, it is possible to conduct approximately unbiased (AU) topology tests to determine whether the data have sufficient information to reject alternative phylogenetic hypotheses using an alpha-level of 0.05 as the significance threshold. The hypotheses tested are shown in Table 1 and include tests for the monophyly of eukaryote sequences as a whole, the grouping of *A. castellanii* sequences with homologs from other amoebozoans, and tests for grouping of eukaryotic and/or *A. castellanii* sequences with α-proteobacterial sequences (as expected if they were of mitochondrial origin). These tests show, for example, that monophyly of the *A. castellanii* sequence with other amoebozoan homologs, such as the sequence from *Mastigamoeba balamuthi* or the *Entamoeba histolytica* sequence, can be rejected, whereas eukaryote monophyly itself cannot be rejected. Preliminary RAxML and FastTree analyses recovered one unusually long-branching eukaryotic/bacterial clade, corresponding to the major [FeFe]-hydrogenase Clade B described in Hug et al. [16], which includes the *M. balamuthi*, both *Trimastix pyriformis*, and one of the *E. histolytica* enzymes. Separate analyses were performed excluding the taxa in this clade (Figure S5). Removing this clade did not alter the overall topology enough to recover eukaryote monophyly; again, however, in AU tests using this dataset, eukaryote monophyly was not rejected.

**Table 1:** Approximately unbiased (AU) tests of alternate topologies.

Hypothesis tested	AU testP-value
[FeFe]-hydrogenase	
ML tree	0.952
Eukaryote monophyly	0.094
*Acanthamoeba*+*Mastigamoeba* monophyly	3e-41
*Acanthamoeba*+short-branching *Entamoeba* monophyly	4e-06
*Acanthamoeba*+long-branching *Entamoeba* monophyly	0.004
(*Acanthamoeba*, α-prot.s^1^), other eukaryotes, (bacteria)	0.015
[FeFe]-hydrogenase (long branches removed)	
ML tree	0.921
Eukaryote monophyly	0.547
*Acanthamoeba*+*Entamoeba* monophyly	0.003
(*Acanthamoeba*, α-prot.s^1^), other eukaryotes, (bacteria)	2e-05
PFO	
ML tree	0.707
Eukaryote monophyly	0.674
*Acanthamoeba*+*Mastigamoeba* monophyly	0.181
*Acanthamoeba*+*Entamoeba* monophyly	0.059
*Acanthamoeba*+*Mastigamoeba*+*Entamoeba* monophyly	0.045
(*Acanthamoeba*, α-prot.s^1^), other eukaryotes, (bacteria)	2e-37
ASCT	
ML tree	0.803
Eukaryote monophyly	0.002
(*Acanthamoeba*, Euk clade 1^2^), other eukaryotes (bacteria)	0.625
(*Acanthamoeba*, *Malawimonas*, *Capsaspora*, Euk clade 1^2^), other euks, (bacteria)	0.452
(Euk clade 1^2^, α-prot.s^1^), other eukaryotes *Pseudovibrio Commensalibact.*, (bacteria)	0.036
(Euk clade 2^3^, α-prot.s^1^), other eukaryotes, *Pseudovibrio*, *Commensalibact.*, (bacteria)	0.032
(*Acanthamoeba*, α-prot.s^1^), other eukaryotes, *Pseudovibrio*, *Commensalibacter*, (bacteria)	0.032
HydE	
ML tree	0.848
Eukaryote monophyly	0.866
*Acanthamoeba*+*Mastigamoeba* monophyly	0.948
((Eukaryotes, α-prot.s^1^), β-prot.s^4^), *Spironucleus*, (bacteria)	0.605
HydF	
ML tree	0.757
Eukaryote monophyly	0.758
*Acanthamoeba*+*Mastigamoeba* monophyly	0.678
HydG	
ML tree	0.985
Eukaryote monophyly	0.944
*Acanthamoeba*+*Mastigamoeba* monophyly	0.880

1 α-proteobacteria.

2 Euk clade 1: opisthokonts+*Blastocystis*.

3 Euk clade 2: Thecamonas+Salpingoeca+Monosiga.

4 β-proteobacteria.

The only previous phylogenetic analysis of [FeFe]-hydrogenase maturases [16] was notable in that it recovered eukaryote monophyly for all three enzymes, despite this not having been the case for [FeFe]-hydrogenase itself – even accounting for the lack of known sequences of these enzymes in some eukaryotes. This observation also holds true for our analyses (Figures S6,S7, S8), despite the additional eukaryotic sequence data that have become available in the interim. Although *Spironucleus vortens* groups with α- and β-proteobacteria in the HydE tree (Figure S6), this position has low bootstrap support and, as with the other two maturases, topology tests (Table 1) do not reject eukaryote monophyly for HydE. In contrast with the topology tests for [FeFe]-hydrogenase, PFO and ASCT1B, a specific grouping of eukaryotes and α-proteobacteria (as expected if the enzymes were of mitochondrial origin) is not rejected by topology tests (Table 1). Within the main eukaryotic clade, low support precludes drawing conclusions about internal relationships, including that of *A. castellanii*; monophyly of *A. castellanii* and *M. balamuthi* is not rejected by topology tests for any of the maturases.

**Figure 5:**
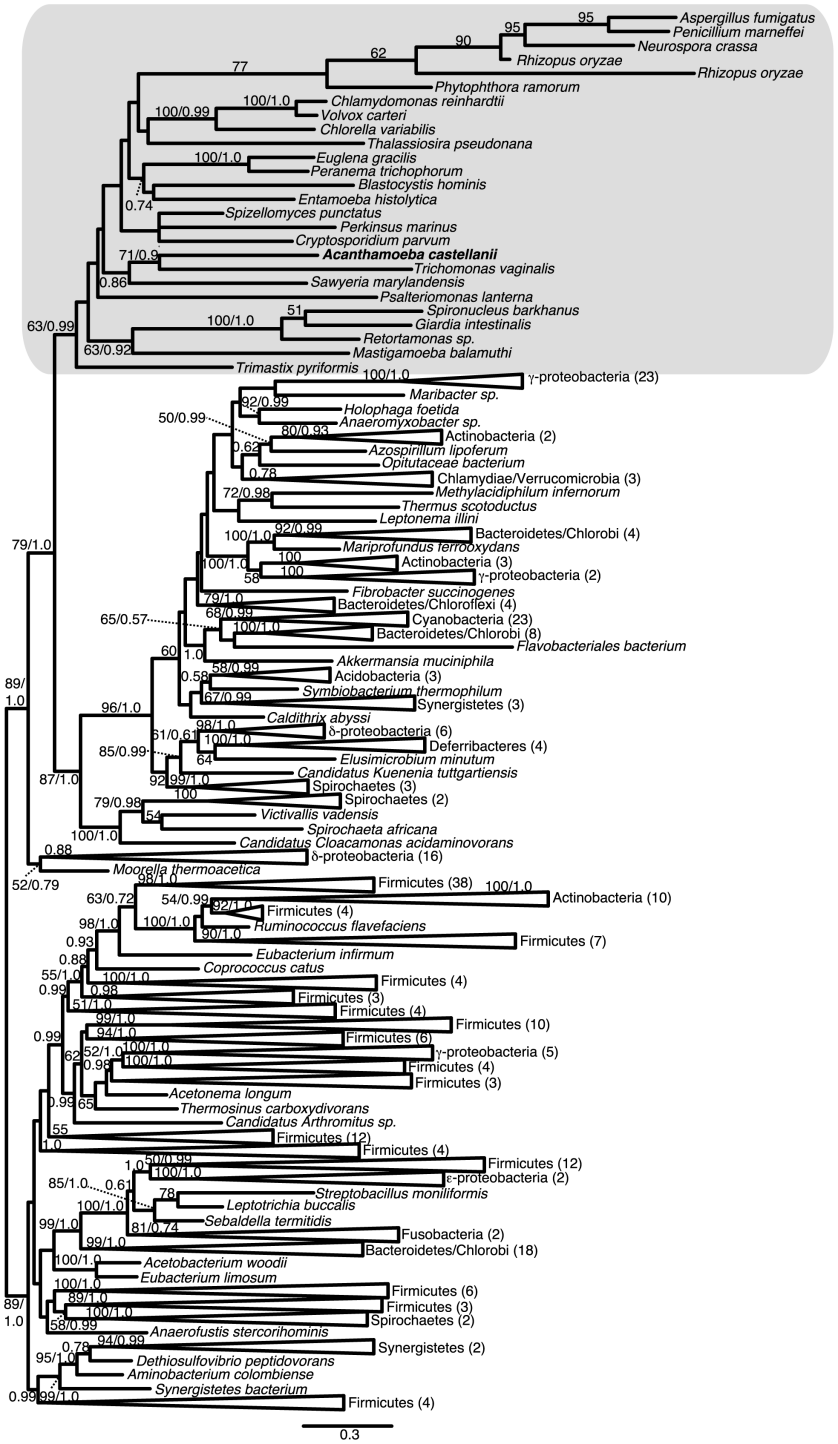
Phylogeny of PFO in eukaryotes and bacteria. The topology shown is the ML tree estimated by RAxML; 954 sites were examined across 335 taxa. Bootstrap support 50% and posterior probabilities ≥0.5 are shown. Eukaryotes are shaded gray.

Previous phylogenetic analyses of PFO reached different conclusions as to the recovery of eukaryote monophyly [16], [34]. Our analyses excluded a long-branching *Monocercomonoides* sequence that may have distorted the topology recovered by Hug et al. [16]; consequently, we recover eukaryotic monophyly (Figure 5), a finding consistent with that of Horner and colleagues [34]. As in the case of [FeFe]-hydrogenase, a hypothetical *Acanthamoeba*+*Entamoeba* clade was rejected by topology tests; but an *Acanthamoeba*+*Mastigamoeba* clade was not. However, large groupings within the eukaryote clade have poor support, preventing the inference of clear internal relationships.

A previous neighbor-net analysis of ASCT1B and ASCT1C sequences [23] recovered a monophyletic cluster of animal sequences, representing the only eukaryotes known to possess ASCT1B-like sequences at that time. Our analyses include additional animal sequences, as well as sequences from a number of other lineages. Monophyly of all of these eukaryote sequences is rejected in topology tests (Table 1), as is the grouping of α-proteobacteria with any of the major eukaryote groups. However, both the grouping of *A. castellanii* with the aerobic flagellate *Malawimonas jakobiformis* as well as the branches separating this clade from away from opisthokonts and *Blastocystis* have low bootstrap support (Figure S9), and an alternate position of this organism, grouping with opisthokonts and *Blastocystis*, is not rejected.

## Discussion

**Figure 6:**
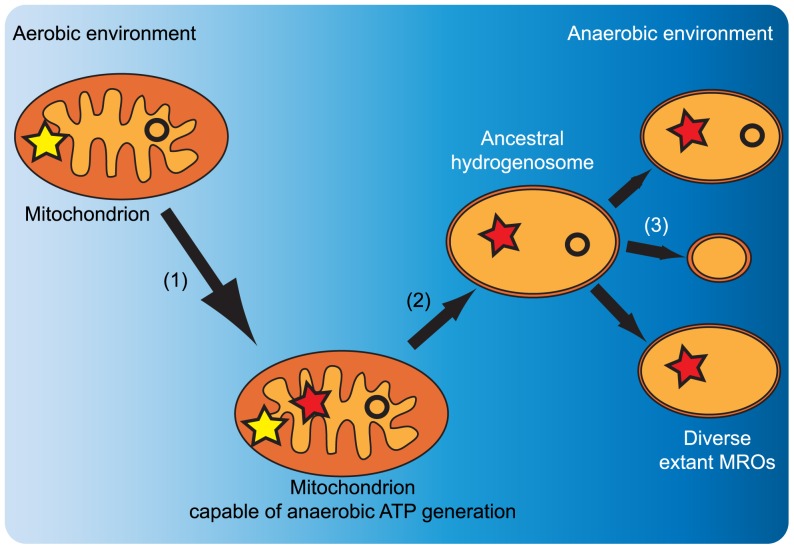
The origins of mitochondrion-related organelles. A hypothetical scenario for the acquisition of anaerobic ATP generation enzymes and the subsequent emergence of extant mitochondrion-related organelles (MROs). (1) Acquisition of anaerobic energy generation enzymes. (2) Loss of the capacity for oxidative phosphorylation. (3) Loss of diverse mitochondrial functions. Yellow stars represent the electron transport chain, while red stars represent the hydrogenosomal anaerobic ATP generation pathway. Circles represent the mitochondrial genomes.

*Acanthamoeba castellanii* is known to inhabit aerobic environments and to produce energy via oxidative phosphorylation; it possesses a mitochondrion with a functional TCA cycle and electron transport chain similar to those found in other aerobic eukaryotes [49], [70]. Nevertheless, it inhabits a wide range of soil, aquatic, and man-made environments, and it is likely that it encounters low-oxygen conditions with some frequency. An organism with such a lifestyle would likely derive significant survival benefit from being able to function under a wide range of conditions, including periods of anoxia. Here, we present the first case of a complete hydrogenosome-like ATP generation pathway with predicted mitochondrial targeting in a habitually aerobic eukaryote, and show that several of its key enzymes are present in the mitochondria. Our work provides the first evidence supported by localization data for a mitochondrion possessing the metabolic components of a ‘hybrid’ organelle, which may enable it to adopt functions in oxidative phosphorylation or anaerobic metabolism according to the conditions that it encounters. The existence of such organelles in an extant organism immediately suggests a possible sequence of events in the first steps leading to the emergence of hydrogenosomes. Upon acquiring an anaerobic energy-generating pathway, a previously obligate aerobe would be able to thrive in a more diverse range of habitats, as well as surviving temporal fluctuations in oxygen levels. Subsequently, descendents of such a cell inhabiting exclusively low-oxygen environments, with a reduced need to perform oxidative phosphorylation, might lose components of the electron transport chain, as well as other mitochondrial functions (Figure 6).

We have confirmed the presence in *A. castellanii* of a complete anaerobic ATP generation pathway similar to that found in hydrogenosomes. All of these enzymes are predicted to have mitochondrial localization, and we confirm this localization for the characteristic hydrogenosomal energy enzymes PFO, ASCT1B, HydF and [FeFe]-hydrogenase. It should be noted that we cannot exclude the possibility of a dual mitochondrial and cytosolic localization of these enzymes; nevertheless, the presence in mitochondrial fractions of PFO, ASCT1B and HydF, and the elevated localization of [FeFe]-hydrogenase within the mitochondria, suggests that these organelles can function as a site of anaerobic respiration in *A. castellanii*. Further research should elucidate the conditions under which this pathway is induced in *A. castellanii*, and the interplay between anaerobic respiration and encystation as different – or perhaps complementary – survival modes in this organism. In addition, the detection of PFO, ASCT1B and HydF peptides in mitochondrial fractions purified from aerobically grown cells raises the possibility that some or all of these enzymes may be upregulated in response to environmental factors other than oxygen concentration.

The most intriguing question raised by this study concerns the origin of these genes. The physical proximity of [FeFe]-hydrogenase and its three maturases in the genome might suggest the lateral transfer of a single bacterial operon as an acquisition mechanism. However, the presence of so many interspersing genes and the absence of a clear bacterial donor candidate argue against a single, recent transfer from bacteria. Topology tests reject the grouping of *A. castellanii* and α-proteobacteria (with remaining eukaryotes unconstrained) for [FeFe]-hydrogenase, PFO and ASCT1B. This result is consistent with previous studies, which recover at least two independent origins for [FeFe]-hydrogenase [16], [30], [32], [33], [69], and specifically reject α-proteobacterial ancestry for [FeFe]-hydrogenase in topology tests [16].

Eukaryote monophyly is recovered for two of the maturases and for PFO, and is not rejected in topology tests for [FeFe]-hydrogenase and the remaining maturase. This finding might seem consistent with the hydrogen hypothesis, which holds that the original endosymbiont that gave rise to mitochondrion-related organelles within eukaryotes was a facultative anaerobe possessing an [FeFe]-hydrogenase, retained by a methanogenic, hydrogen-dependent host for the hydrogen it generated as a waste product [71]. Nevertheless, the lack of a clear affinity to α-proteobacterial homologues for these enzymes, and their distribution within eukaryotes – in particular their absence from so many taxa closely related to anaerobes – weakens such a conclusion. All or most of these genes might still have been present in the protomitochondrial endosymbiont as a result of a lateral gene transfer (LGT) event from a different prokaryote, or might have been acquired by the ancestral eukaryote by other means [36]; these scenarios would be more consistent with the very small number of homologs reported in contemporary α-proteobacteria. However, if this is the case, then (1) the patchy distribution of anaerobic ATP generation enzymes among eukaryotes in general, (2) the rejection of monophyly of *A. castellanii* with two other amoebozoans for [FeFe]-hydrogenase, and (3) the absence of genes for these enzymes in the genomes of other members of Amoebozoa, remain to be explained.

Intriguingly, the existence of monophyletic eukaryotic clades with unusual internal topology has been reported for other enzymes with patchy distributions among eukaryotes. The authors of these studies proposed multiple lateral transfers between eukaryotes as a hypothesis to explain patchy distributions and unexpected phylogenetic relationships for other enzymes found in both aerobic and anaerobic protists [13], [72]–[76]. This mode of acquisition would provide an attractive alternative scenario for the acquisition of anaerobic metabolism genes; the transfer of genes between eukaryotes would remove the need for the acquisition of eukaryotic regulatory sequences for the enzymes in the recipient, and would account for the distribution of anaerobic ATP generation enzymes in extant eukaryotes. Furthermore, it would provide an elegant explanation for the common pool of anaerobic ATP generation enzymes found in anaerobic eukaryotes [7]. Frustratingly, the lack of phylogenetic resolution in much of the tree, including for internal eukaryote relationships and for the position of *A. castellanii* itself, does not allow us to draw strong inferences that would help us to distinguish among competing hypotheses as to the origin of anaerobic enzymes in *A. castellanii*.

Our work cements the possibility, initially raised by Hug et al. [16], that anaerobic ATP generation enzymes might be more widespread among eukaryotes than previously thought, not being limited to anaerobic or microaerobic lineages. It also highlights the importance of exploratory sequencing efforts focusing on a wide range of organisms. So far, anaerobic energy enzymes have been described in two non-photosynthetic organisms, *A. castellanii* and *N. gruberi*. As an opportunistic human pathogen that also harbours pathogenic bacteria, and a close relative of an opportunistic human pathogen, respectively, both of these organisms have links to human health, making them attractive targets for sequencing efforts. However, the more important link between them is likely their lifestyle, which exposes them to a wide range of habitats that vary in oxygen concentration, a lifestyle likely made possible by the anaerobic energy enzymes they have acquired. Free-living soil protists are relatively poorly studied, and investigations into the metabolic complements of a wider range of such organisms will reveal whether these hybrid organelles are found more commonly in nature than previously suspected.

## Supporting Information

Methods S1 **Methods relating to results shown in Figures S2–S5.**
(DOCX)

Figure S1 **Assessment of mitochondrial purity by comparing rRNA profiles of total cellular RNA and total mitochondrial RNA from** ***A. castellanii*** **.** A) An EtBr-stained 6% (w:v) acrylamide, 7 M urea gel loaded with varying quantities of total cellular (T) and total mitochondrial (M) RNA. Numbers are quantities of RNA (in µg) loaded in each lane. The profiles of large rRNA species in T and M are distinct, suggesting that the mitochondrial fraction is relatively free of contaminating cytosolic rRNA (and presumably cytosolic ribosomal proteins). *A. castellanii* cytosolic (c) and mitochondrial (m) LSU and SSU species are identified (note that the cLSU is split). B) 10% acrylamide gel as in A). Note that a mitochondrial 5S rRNA (m5S) is visualized in the M lane and, to a much lower extent, in the T lane. A band corresponding in size to cytosolic 5.8S rRNA is visible in an overloaded M lane; however, no cytosolic 5S (c5S) is detectable.
(TIF)

Figure S2 **Western blot showing recognition of the recombinant [FeFe]-hydrogenase by the homologous anti-[FeFe]-hydrogenase antibody.** Lanes 1, 3, 5 and 7: inclusion bodies from C41(DE) cells expressing empty pET-16b vector. Lanes 2, 4, 6 and 8: inclusion bodies from C41(DE) cells expressing recombinant *A. castellanii* [FeFe]-hydrogenase from pET-16b. Lanes 1 and 2: anti-His-tag antibody, exposed for a shorter period of time than lanes 3–8 in order to avoid overexposure. Lanes 3 and 4: anti [FeFe]-hydrogenase antibody only. Lanes 5 and 6: anti-[FeFe]-hydrogenase antibody incubated with inclusion bodies from C41(DE) cells expression empty pET-16b. Lanes 7 and 8: anti-[FeFe]-hydrogenase antibody incubated with inclusion bodies from C41(DE) cells expressing recombinant *A. castellanii* [FeFe]-hydrogenase from pET-16b.
(TIF)

Figure S3 **Immunogold localization of [FeFe]-hydrogenase in** ***A. castellanii*** **trophozoites exposed to anaerobic conditions for 24 hr.** A. Whole cell fixed for immunogold staining; scale bar, 500 nm; this image has been cropped in order to show only the whole cell from which the insets shown were derived. B. and C. Magnified sections from the cell depicted in (A), showing gold particles corresponding to [FeFe]-hydrogenase localization; scale bar, 500 nm. D. Mean density of immunogold labeling in the cytosol, nucleus and mitochondria (8 cells). Brightness and contrast have been adjusted in each image to enhance visibility of the mitochondria and gold particles.
(TIF)

Figure S4 **Immunogold localization of [FeFe]-hydrogenase in** ***A. castellanii*** **trophozoites exposed to anaerobic conditions for 6 hr.** A. Whole cell fixed for immunogold staining; scale bar, 500 nm; this image has been cropped in order to show only the whole cell from which the insets shown were derived. B. and C. Magnified sections from the cell depicted in (A), showing gold particles corresponding to [FeFe]-hydrogenase localization; scale bar, 500 nm. D. Mean density of immunogold labeling in the cytosol, nucleus and mitochondria (7 cells). Brightness and contrast have been adjusted in each image to enhance visibility of the mitochondria and gold particles.
(TIF)

Figure S5 **Phylogeny of [FeFe]-hydrogenase in eukaryotes and bacteria excluding long-branching taxa.** Taxa forming a long-branching clade, corresponding to Clade B identified by Hug et al. (2010), have been excluded from these analyses. The topology shown is the ML tree generated by RAxML analyses; 328 sites were examined across 151 taxa. Bootstrap support values ≥50% and posterior probabilities ≥0.5 are shown. Eukaryotes are shaded gray.
(TIF)

Figure S6 **Phylogeny of HydE in eukaryotes and bacteria.** The topology shown is the ML tree generated by RAxML analyses; 264 sites were examined across 109 taxa. Bootstrap support values ≥50% and posterior probabilities ≥0.5 are shown. Eukaryotes are shaded gray.
(TIF)

Figure S7 **Phylogeny of HydF in eukaryotes and bacteria.** The topology shown is the ML tree generated by RAxML analyses; 307 sites were examined across 196 taxa. Bootstrap support values ≥50% and posterior probabilities ≥0.5 are shown. Eukaryotes are shaded gray.
(TIF)

Figure S8 **Phylogeny of HydG in eukaryotes and bacteria.** The topology shown is the ML tree generated by RAxML analyses. 391 sites were examined across 87 taxa; bootstrap support values greater than or equal to 50%, and posterior probabilities greater than or equal to 0.5, are shown. Eukaryotes are shaded gray.
(TIF)

Figure S9 **Phylogeny of ASCT1B in eukaryotes and bacteria.** The topology shown is the ML tree generated by RAxML analyses; 315 sites were examined across 221 taxa. Bootstrap support values ≥50% and posterior probabilities ≥0.5 are shown. Eukaryotes are shaded gray.
(TIF)

Table S1 **Accession numbers of genes and ESTs encoding anaerobic energy generation enzymes.**
(DOCX)

## References

[1] Tsaousis AD, Leger MM, Stairs CAW, Roger AJ (2012) The biochemical adaptations of mitochondrion-related organelles of parasitic and free-living microbial eukaryotes to low oxygen environments. In: Altenbach AV, Bernhard JM, Seckbach J, editors. Anoxia: Evidence for Eukaryote Survival and Paleontological Strategies. Dordrecht: Springer. pp. 51–81.

[2] Lindmark DG, Muller M (1973) Hydrogenosome, a cytoplasmic organelle of the anaerobic flagellate *Tritrichomonas foetus*, and its role in pyruvate metabolism. J Biol Chem 248: 7724–7728. 4750424

[3] Dyall SD, Yan W, Delgadillo-Correa MG, Lunceford A, Loo JA, et al (2004) Non-mitochondrial complex I proteins in a hydrogenosomal oxidoreductase complex. Nature 431: 1103–1107. 15510149 10.1038/nature02990

[4] Hrdý I, Hirt RP, Dolezal P, Bardonova L, Foster PG, et al (2004) *Trichomonas* hydrogenosomes contain the NADH dehydrogenase module of mitochondrial complex I. Nature 432: 618–622. 15577909 10.1038/nature03149

[5] Ellis JE, Williams R, Cole D, Cammack R, Lloyd D (1993) Electron transport components of the parasitic protozoon *Giardia lamblia* . FEBS Lett 325: 196–200. 8391475 10.1016/0014-5793(93)81072-8

[6] Rodriguez MA, Hidalgo ME, Sanchez T, Orozco E (1996) Cloning and characterization of the *Entamoeba histolytica* pyruvate: ferredoxin oxidoreductase gene. Mol Biochem Parasitol 78: 273–277. 8813698 10.1016/s0166-6851(96)02613-8

[7] Müller M, Mentel M, van Hellemond JJ, Henze K, Woehle C, et al (2012) Biochemistry and evolution of anaerobic energy metabolism in eukaryotes. Microbiol Mol Biol Rev 76: 444–495. 22688819 10.1128/MMBR.05024-11 PMC3372258

[8] Steinbuchel A, Muller M (1986) Anaerobic pyruvate metabolism of *Tritrichomonas foetus* and *Trichomonas vaginalis* hydrogenosomes. Mol Biochem Parasitol 20: 57–65. 3090435 10.1016/0166-6851(86)90142-8

[9] Hrdý I, Müller M (1995) Primary structure and eubacterial relationships of the pyruvate:ferredoxin oxidoreductase of the amitochondriate eukaryote *Trichomonas vaginalis* . J Mol Evol 41: 388–396. 7563125

[10] Ctrnacta V, Ault JG, Stejskal F, Keithly JS (2006) Localization of pyruvate:NADP+ oxidoreductase in sporozoites of *Cryptosporidium parvum* . J Eukaryot Microbiol 53: 225–231. 16872290 10.1111/j.1550-7408.2006.00099.x

[11] Buetow DE (1989) The mitochondrion. In: Buetow DE, editor. The Biology of Euglena, Vol 4, Subcellular Biochemistry and Molecular-Biology. San Diego: Academic Press. pp. 247–314.

[12] Gelius-Dietrich G, Henze K (2004) Pyruvate formate lyase (PFL) and PFL activating enzyme in the chytrid fungus *Neocallimastix frontalis*: a free-radical enzyme system conserved across divergent eukaryotic lineages. J Eukaryot Microbiol 51: 456–463. 15352329 10.1111/j.1550-7408.2004.tb00394.x

[13] Stairs CW, Roger AJ, Hampl V (2011) Eukaryotic pyruvate formate lyase and its activating enzyme were acquired laterally from a firmicute. Molecular Biology and Evolution 28: 2087–2099. 21293046 10.1093/molbev/msr032

[14] Posewitz MC, King PW, Smolinski SL, Zhang L, Seibert M, et al (2004) Discovery of two novel radical S-adenosylmethionine proteins required for the assembly of an active [Fe] hydrogenase. J Biol Chem 279: 25711–25720. 15082711 10.1074/jbc.M403206200

[15] Pütz S, Dolezal P, Gelius-Dietrich G, Bohacova L, Tachezy J, et al (2006) Fe-hydrogenase maturases in the hydrogenosomes of *Trichomonas vaginalis* . Eukaryot Cell 5: 579–586. 16524912 10.1128/EC.5.3.579-586.2006 PMC1398061

[16] Hug LA, Stechmann A, Roger AJ (2010) Phylogenetic distributions and histories of proteins involved in anaerobic pyruvate metabolism in eukaryotes. Mol Biol Evol 27: 311–324. 19805439 10.1093/molbev/msp237

[17] Tielens AG, van Grinsven KW, Henze K, van Hellemond JJ, Martin W (2010) Acetate formation in the energy metabolism of parasitic helminths and protists. Int J Parasitol 40: 387–397. 20085767 10.1016/j.ijpara.2009.12.006

[18] Riviere L, van Weelden SW, Glass P, Vegh P, Coustou V, et al (2004) Acetyl:succinate CoA-transferase in procyclic *Trypanosoma brucei*. Gene identification and role in carbohydrate metabolism. J Biol Chem 279: 45337–45346. 15326192 10.1074/jbc.M407513200

[19] Boxma B, de Graaf RM, van der Staay GW, van Alen TA, Ricard G, et al (2005) An anaerobic mitochondrion that produces hydrogen. Nature 434: 74–79. 15744302 10.1038/nature03343

[20] van Grinsven KW, van Hellemond JJ, Tielens AG (2009) Acetate:succinate CoA-transferase in the anaerobic mitochondria of *Fasciola hepatica* . Mol Biochem Parasitol 164: 74–79. 19103231 10.1016/j.molbiopara.2008.11.008

[21] Oulton MM, Amons R, Liang P, MacRae TH (2003) A 49 kDa microtubule cross-linking protein from *Artemia franciscana* is a coenzyme A-transferase. Eur J Biochem 270: 4962–4972. 14653822 10.1046/j.1432-1033.2003.03898.x

[22] Fritz-Laylin LK, Prochnik SE, Ginger ML, Dacks JB, Carpenter ML, et al (2010) The genome of *Naegleria gruberi* illuminates early eukaryotic versatility. Cell 140: 631–642. 20211133 10.1016/j.cell.2010.01.032

[23] van Grinsven KW, Rosnowsky S, van Weelden SW, Putz S, van der Giezen M, et al (2008) Acetate:succinate CoA-transferase in the hydrogenosomes of *Trichomonas vaginalis*: identification and characterization. J Biol Chem 283: 1411–1418. 18024431 10.1074/jbc.M702528200

[24] Lantsman Y, Tan KS, Morada M, Yarlett N (2008) Biochemical characterization of a mitochondrial-like organelle from *Blastocystis* sp. subtype 7. Microbiology 154: 2757–2766. 18757809 10.1099/mic.0.2008/017897-0

[25] Stechmann A, Hamblin K, Perez-Brocal V, Gaston D, Richmond GS, et al (2008) Organelles in *Blastocystis* that blur the distinction between mitochondria and hydrogenosomes. Curr Biol 18: 580–585. 18403202 10.1016/j.cub.2008.03.037 PMC2428068

[26] Marvin-Sikkema FD, Pedro Gomes TM, Grivet JP, Gottschal JC, Prins RA (1993) Characterization of hydrogenosomes and their role in glucose metabolism of *Neocallimastix* sp. L2. Arch Microbiol 160: 388–396. 8257282 10.1007/BF00252226

[27] Gaffron H, Rubin J (1942) Fermentative and photochemical production of hydrogen in algae. J Gen Physiol 26: 219–240. 19873339 10.1085/jgp.26.2.219 PMC2142062

[28] Happe T, Kaminski A (2002) Differential regulation of the Fe-hydrogenase during anaerobic adaptation in the green alga *Chlamydomonas reinhardtii* . Eur J Biochem 269: 1022–1032. 11846805 10.1046/j.0014-2956.2001.02743.x

[29] Forestier M, King P, Zhang L, Posewitz M, Schwarzer S, et al (2003) Expression of two [Fe]-hydrogenases in *Chlamydomonas reinhardtii* under anaerobic conditions. Eur J Biochem 270: 2750–2758. 12823545 10.1046/j.1432-1033.2003.03656

[30] Horner DS, Heil B, Happe T, Embley TM (2002) Iron hydrogenases–ancient enzymes in modern eukaryotes. Trends Biochem Sci 27: 148–153. 11893512 10.1016/s0968-0004(01)02053-9

[31] Hackstein J (2005) Eukaryotic Fe-hydrogenases—old eukaryotic heritage or adaptive acquisitions. Biochem Soc Trans 33: 47–50. 15667261 10.1042/BST0330047

[32] Meyer J (2007) [FeFe] hydrogenases and their evolution: a genomic perspective. Cell Mol Life Sci 64: 1063–1084. 17353991 10.1007/s00018-007-6477-4 PMC11136429

[33] Vignais PM, Billoud B (2007) Occurrence, classification, and biological function of hydrogenases: an overview. Chem Rev 107: 4206–4272. 17927159 10.1021/cr050196r

[34] Horner DS, Hirt RP, Embley TM (1999) A single eubacterial origin of eukaryotic pyruvate: ferredoxin oxidoreductase genes: implications for the evolution of anaerobic eukaryotes. Mol Biol Evol 16: 1280–1291. 10486982 10.1093/oxfordjournals.molbev.a026218

[35] Rotte C, Stejskal F, Zhu G, Keithly J, Martin W (2001) Pyruvate: NADP oxidoreductase from the mitochondrion of *Euglena gracilis* and from the apicomplexan *Cryptosporidium parvum*: a biochemical relic linking pyruvate metabolism in mitochondriate and amitochondriate protists. Molecular Biology and Evolution 18: 710. 11319255 10.1093/oxfordjournals.molbev.a003853

[36] Embley TM (2006) Multiple secondary origins of the anaerobic lifestyle in eukaryotes. Philos Trans R Soc Lond B Biol Sci 361: 1055–1067. 16754614 10.1098/rstb.2006.1844 PMC1578728

[37] Marciano-Cabral F, Cabral G (2003) *Acanthamoeba* spp. as agents of disease in humans. Clinical Microbiology Reviews 16: 273–307. 12692099 10.1128/CMR.16.2.273-307.2003 PMC153146

[38] Cometá I, Schatz S, Trzyna W, Rogerson A (2011) Tolerance of naked amoebae to low oxygen levels with an emphasis on the genus *Acanthamoeba* . Acta Protozoologica 50: 33–40.

[39] Turner NA, Biagini GA, Lloyd D (1997) Anaerobiosis-induced differentiation of *Acanthamoeba castellanii* . Fems Microbiology Letters 157: 149–153.

[40] Huang X, Madan A (1999) CAP3: A DNA sequence assembly program. Genome Res 9: 868–877. 10508846 10.1101/gr.9.9.868 PMC310812

[41] Chevreux B, Wetter T, Suhai S (1999) Genome sequence assembly using trace signals and additional sequence information. Computer Science and Biology: Proceedings of the German Conference on Bioinformatics 99: 45–56.

[42] Chevreux B, Pfisterer T, Drescher B, Driesel AJ, Müller WEG, et al (2004) Using the miraEST assembler for reliable and automated mRNA transcript assembly and SNP detection in sequenced ESTs. Genome Research 14: 1147–1159. 15140833 10.1101/gr.1917404 PMC419793

[43] Altschul SF, Madden TL, Schaffer AA, Zhang J, Zhang Z, et al (1997) Gapped BLAST and PSI-BLAST: a new generation of protein database search programs. Nucleic Acids Res 25: 3389–3402. 9254694 10.1093/nar/25.17.3389 PMC146917

[44] O'Brien EA, Koski LB, Zhang Y, Yang L, Wang E, et al (2007) TBestDB: a taxonomically broad database of expressed sequence tags (ESTs). Nucleic Acids Res 35: D445–451. 17202165 10.1093/nar/gkl770 PMC1899108

[45] Nielsen H, Engelbrecht J, Brunak S, von Heijne G (1997) Identification of prokaryotic and eukaryotic signal peptides and prediction of their cleavage sites. Protein Engineering 10: 1–6. 10.1093/protein/10.1.1 9051728

[46] Emanuelsson O, Nielsen H, Brunak S, von Heijne G (2000) Predicting subcellular localization of proteins based on their N-terminal amino acid sequence. J Mol Biol 300: 1005–1016. 10891285 10.1006/jmbi.2000.3903

[47] Emanuelsson O, Brunak S, von Heijne G, Nielsen H (2007) Locating proteins in the cell using TargetP, SignalP and related tools. Nat Protoc 2: 953–971. 17446895 10.1038/nprot.2007.131

[48] Lohan AJ, Gray MW (2007) Analysis of 5′- or 3′-Terminal tRNA Editing: Mitochondrial 5′ tRNA Editing in *Acanthamoeba castellanii* as the Exemplar. Meth Enzymol 424: 221–242. 10.1016/S0076-6879(07)24010-8 17662843

[49] Gawryluk RM, Chisholm KA, Pinto DM, Gray MW (2012) Composition of the mitochondrial electron transport chain in *Acanthamoeba castellanii*: structural and evolutionary insights. Biochim Biophys Acta 1817: 2027–2037. 22709906 10.1016/j.bbabio.2012.06.005

[50] Perkins DN, Pappin DJ, Creasy DM, Cottrell JS (1999) Probability-based protein identification by searching sequence databases using mass spectrometry data. Electrophoresis 20: 3551–3567. 10612281 10.1002/(SICI)1522-2683(19991201)20:18<3551::AID-ELPS3551>3.0.CO;2-2

[51] Gawryluk RM, Gray MW (2010) Evidence for an early evolutionary emergence of gamma-type carbonic anhydrases as components of mitochondrial respiratory complex I. BMC Evol Biol 10: 176. 20546574 10.1186/1471-2148-10-176 PMC2900272

[52] Edgar RC (2004) MUSCLE: a multiple sequence alignment method with reduced time and space complexity. BMC Bioinformatics 5: 113. 15318951 10.1186/1471-2105-5-113 PMC517706

[53] Edgar RC (2004) MUSCLE: multiple sequence alignment with high accuracy and high throughput. Nucleic Acids Res 32: 1792–1797. 15034147 10.1093/nar/gkh340 PMC390337

[54] Bradley RK, Roberts A, Smoot M, Juvekar S, Do J, et al (2009) Fast statistical alignment. PLoS Comput Biol 5: e1000392. 19478997 10.1371/journal.pcbi.1000392 PMC2684580

[55] Katoh K, Misawa K, Kuma K, Miyata T (2002) MAFFT: a novel method for rapid multiple sequence alignment based on fast Fourier transform. Nucleic Acids Res 30: 3059–3066. 12136088 10.1093/nar/gkf436 PMC135756

[56] Katoh K, Kuma K, Toh H, Miyata T (2005) MAFFT version 5: improvement in accuracy of multiple sequence alignment. Nucleic Acids Res 33: 511–518. 15661851 10.1093/nar/gki198 PMC548345

[57] Katoh K, Toh H (2008) Recent developments in the MAFFT multiple sequence alignment program. Brief Bioinform 9: 286–298. 18372315 10.1093/bib/bbn013

[58] Criscuolo A, Gribaldo S (2010) BMGE (Block Mapping and Gathering with Entropy): a new software for selection of phylogenetic informative regions from multiple sequence alignments. BMC Evol Biol 10: 210. 20626897 10.1186/1471-2148-10-210 PMC3017758

[59] Price MN, Dehal PS, Arkin AP (2009) FastTree: computing large minimum evolution trees with profiles instead of a distance matrix. Mol Biol Evol 26: 1641–1650. 19377059 10.1093/molbev/msp077 PMC2693737

[60] Stamatakis A (2006) RAxML-VI-HPC: maximum likelihood-based phylogenetic analyses with thousands of taxa and mixed models. Bioinformatics 22: 2688–2690. 16928733 10.1093/bioinformatics/btl446

[61] Le SQ, Gascuel O (2008) An improved general amino acid replacement matrix. Mol Biol Evol 25: 1307–1320. 18367465 10.1093/molbev/msn067

[62] Lartillot N, Lepage T, Blanquart S (2009) PhyloBayes 3: a Bayesian software package for phylogenetic reconstruction and molecular dating. Bioinformatics 25: 2286–2288. 19535536 10.1093/bioinformatics/btp368

[63] Le SQ, Lartillot N, Gascuel O (2008) Phylogenetic mixture models for proteins. Philos Trans R Soc Lond B Biol Sci 363: 3965–3976. 18852096 10.1098/rstb.2008.0180 PMC2607422

[64] Shimodaira H, Hasegawa M (2001) CONSEL: for assessing the confidence of phylogenetic tree selection. Bioinformatics 17: 1246–1247. 11751242 10.1093/bioinformatics/17.12.1246

[65] Gaudet P, Fey P, Basu S, Bushmanova YA, Dodson R, et al (2011) dictyBase update 2011: web 2.0 functionality and the initial steps towards a genome portal for the Amoebozoa. Nucleic Acids Res 39: D620–624. 21087999 10.1093/nar/gkq1103 PMC3013695

[66] Schneider G, Sjoling S, Wallin E, Wrede P, Glaser E, et al (1998) Feature-extraction from endopeptidase cleavage sites in mitochondrial targeting peptides. Proteins 30: 49–60. 9443340

[67] Williams K, Lowe PN, Leadlay PF (1987) Purification and characterization of pyruvate: ferredoxin oxidoreductase from the anaerobic protozoon *Trichomonas vaginalis* . Biochem J 246: 529–536. 3500709 10.1042/bj2460529 PMC1148305

[68] Horner DS, Foster PG, Embley TM (2000) Iron hydrogenases and the evolution of anaerobic eukaryotes. Mol Biol Evol 17: 1695–1709. 11070057 10.1093/oxfordjournals.molbev.a026268

[69] Voncken FG, Boxma B, van Hoek AH, Akhmanova AS, Vogels GD, et al (2002) A hydrogenosomal [Fe]-hydrogenase from the anaerobic chytrid *Neocallimastix* sp. L2. Gene 284: 103–112. 11891051 10.1016/s0378-1119(02)00388-8

[70] Edwards SW, Lloyd D (1978) Properties of mitochondria isolated from cyanide-sensitive and cyanide-stimulated cultures of *Acanthamoeba castellanii* . Biochem J 174: 203–211. 212020 10.1042/bj1740203 PMC1185900

[71] Martin W, Muller M (1998) The hydrogen hypothesis for the first eukaryote. Nature 392: 37–41. 9510246 10.1038/32096

[72] Andersson JO (2009) Horizontal gene transfer between microbial eukaryotes. Methods Mol Biol 532: 473–487. 19271202 10.1007/978-1-60327-853-9_27

[73] Andersson JO (2009) Gene transfer and diversification of microbial eukaryotes. Annu Rev Microbiol 63: 177–193. 19575565 10.1146/annurev.micro.091208.073203

[74] Andersson JO, Sjogren AM, Horner DS, Murphy CA, Dyal PL, et al (2007) A genomic survey of the fish parasite *Spironucleus salmonicida* indicates genomic plasticity among diplomonads and significant lateral gene transfer in eukaryote genome evolution. BMC Genomics 8: 51. 17298675 10.1186/1471-2164-8-51 PMC1805757

[75] Takishita K, Chikaraishi Y, Leger MM, Kim E, Yabuki A, et al (2012) Lateral transfer of tetrahymanol-synthesizing genes has allowed multiple diverse eukaryote lineages to independently adapt to environments without oxygen. Biol Direct 7: 5. 22296756 10.1186/1745-6150-7-5 PMC3317845

[76] Andersson JO, Hirt RP, Foster PG, Roger AJ (2006) Evolution of four gene families with patchy phylogenetic distributions: influx of genes into protist genomes. BMC Evol Biol 6: 27. 16551352 10.1186/1471-2148-6-27 PMC1484493

