# Supplementary material for: Evidence for a Hydrogenosomal-Type Anaerobic ATP Generation Pathway in Acanthamoeba castellanii
Source: PLoS One. 2013 Sep 27;8(9):e69532. doi: 10.1371/journal.pone.0069532 (PMC3785491; doi:10.1371/journal.pone.0069532)
Supplement: Methods S1 — Methods relating to results shown in Figures S2–S5. (DOCX) [file pone.0069532.s001.docx]

**Supporting Methods**

*Anaerobic induction*

Prior to anaerobic induction, Neff base medium, glucose and ferric citrate were placed in an anaerobic chamber (Forma Scientific Anaerobic System model 1024) containing 79.8% N_2_, 10.4% H_2_, 9.8% CO_2_, and allowed to de-gas for 24 hr. High-density *A. castellanii* cells were subjected to centrifugation at 200*g,* and the supernatant was replaced with de-gassed medium, supplemented with glucose, ferric citrate, vitamins and CaCl_2_, inside the anaerobic chamber.

*Antibody production*

[FeFe]-hydrogenase was amplified from cDNA and cloned into pET-16b (Novagen) downstream of a 6xHis tag for recombinant expression in OverExpress™ C41(DE) cells, kindly provided by Prof. John Walker (Medical Research Council Mitochondrial Biology Unit, Cambridge, UK). The recombinant protein was expressed in inclusion bodies, which were purified using BugBuster reagent (Novagen). An antibody against the purified inclusion bodies was raised in rats by GenScript Corporation (Piscataway, NJ, USA).

*Western blotting*

The anti-[FeFe]-hydrogenase antibody was tested against inclusion bodies from C41(DE) cells expressing recombinant *A. castellanii* [FeFe]-hydrogenase from the pET-16b vector, or the empty pET-16b vector. Proteins were blotted onto PVDF membranes and blocked overnight at 4ºC in 5% milk. To remove cross-reaction of the primary antibody, and to confirm the identity of the bound protein, antibody competition assays were performed. The anti-*A. castellanii* [FeFe]-hydrogenase antibody was incubated overnight at 4ºC and for a further 90 min at room temperature the following day with either BugBuster reagent, inclusion bodies from cells expressing empty pET-16b vector in BugBuster reagent, or inclusion bodies from cells expressing recombinant *A. castellanii* [FeFe]-hydrogenase from pET-16b. Following competition, blots were incubated with primary antibody at a ratio of 1:50000 in 1% milk for 1 hr at room temperature, washed, and incubated with horseradish peroxidase-conjugated secondary anti-rat IgG antibody (Sigma) 1:2000 for 1 hr at room temperature, washed again, and incubated with ECL western blotting reagents (Amersham).

*Immunolocalization*

Anaerobically induced cells were subjected to centrifugation at 100*g* for 2 min, and fixed for 1 hr with 4% paraformaldehyde/0.5% glutaraldehyde diluted with 0.1 M sodium cacodylate buffer. In order to prevent the living cells from being exposed to oxygen, all steps up to and including fixation were performed either inside the anaerobic chamber itself, or in containers that had been sealed inside the anaerobic chamber. Fixed cells were rinsed three times for a minimum of 10 min each with 0.1 M sodium cacodylate buffer, and dehydrated with a graduated ethanol series. The dehydrated samples were then embedded in 100% LR White resin and cured for 48 hr in a 60ºC oven. Thin sections were cut using an LKB Huxley ultramicrotome with a diamond knife, and placed onto 300 mesh nickel grids.

Sections were blocked overnight at 4ºC by incubating the grids on droplets of blocking agent (phosphate-buffered saline pH 7.4 containing 0.8% bovine serum albumin and 0.01% Tween 20), and incubated for 3 hr at room temperature on droplets of anti-[FeFe]-hydrogenase primary antibody diluted 1:10 in blocking agent. The grids were washed four times for 10 min each on droplets of blocking agent, then incubated for 1 hr on droplets of gold-conjugated goat anti-rat IgG secondary antibody conjugated to 10 nm gold particles (Sigma; Electron Microscopy Services) diluted 1:20 in blocking agent. Following incubation with the secondary antibody, grids were washed three times for 10 min each in blocking agent, then rinsed three times for 30 sec each in sterile ddH_2_O. Antibody-stained grids were stained for 10 min with 2% aqueous uranyl acetate, rinsed twice with distilled water for 5 min each, stained for 4 min with lead citrate, rinsed, and air-dried. The sections were viewed using a JEOL JEM 1230 transmission electron microscope at 80 kV, and images were captured using a Hamamatsu ORCA-HR digital camera.

The areas of the nucleus, mitochondria and cytosol of each cell cross-section were measured using ImageJ, and the gold particles in each part of the cell were counted by eye.

**Supporting results**

*[FeFe]-hydrogenase* *localizes to the mitochondria*

To experimentally examine the localization of [FeFe]-hydrogenase, the characteristic hydrogenosomal metabolism enzyme, we raised an antibody against recombinant *A. castellanii* [FeFe]-hydrogenase expressed in *E. coli*. The resulting antibody recognized the expressed recombinant enzyme on western blots (Figure S2). Attempts to detect bands in whole cell lysate or crude mitochondrial preparations from *A. castellanii* were unsuccessful (data not shown). This failure is likely attributable to exceptionally low expression levels of this enzyme; the very high concentration of antibody required for localization in immunoelectron microscopy experiments would seem to support this inference. Upon exposure to oxygen, [FeFe]-hydrogenases typically become rapidly and irreversibly inactivated, and are degraded [[67](#_ENREF_67)]. For this reason, we performed immunogold labeling experiments on *A. castellanii* cells that had been exposed to anaerobic conditions for 6 or 24 hr. Antibody staining in these cells was higher in mitochondria than in the cytosol (approx. 2.9-fold) or the nucleus (approx. 1.7-fold), consistent with the presence of a predicted mitochondrial targeting peptide for [FeFe]-hydrogenase (Figures S3, S4). Antibody staining was also enriched in the nucleus compared with the cytosol (approx. 1.8-fold), although to a much lesser extent than in mitochondria. A distant homolog of [FeFe]-hydrogenase, nuclear prelamin A recognition factor (NARF), is localized to the nucleus in some organisms [[68](#_ENREF_68),[69](#_ENREF_69)]; cross-reaction with a nuclear NARF homolog may therefore explain this elevated staining pattern.
