# Supplementary figures and images for: Evidence for a Hydrogenosomal-Type Anaerobic ATP Generation Pathway in Acanthamoeba castellanii
Source: PLoS One. 2013 Sep 27;8(9):e69532. doi: 10.1371/journal.pone.0069532 (PMC3785491; doi:10.1371/journal.pone.0069532)

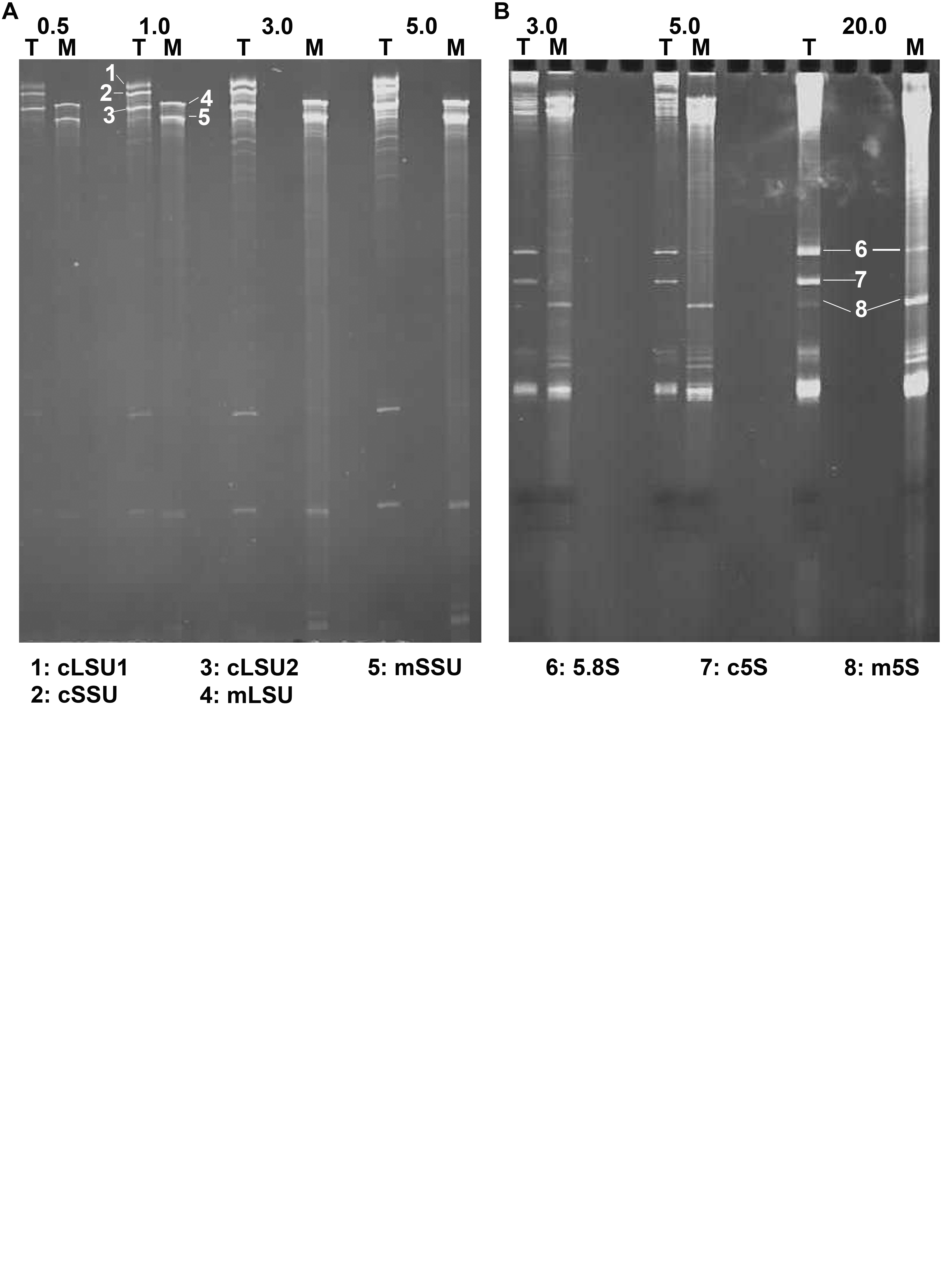

Supplement: Figure S1 — Assessment of mitochondrial purity by comparing rRNA profiles of total cellular RNA and total mitochondrial RNA from A. castellanii . A) An EtBr-stained 6% (w:v) acrylamide, 7 M urea gel loaded with varying quantities of total cellular (T) and total mitochondrial (M) RNA. Numbers are quantities of RNA (in µg) loaded in each lane. The profiles of large rRNA species in T and M are distinct, suggesting that the mitochondrial fraction is relatively free of contaminating cytosolic rRNA (and presumably cytosolic ribosomal proteins). A. castellanii cytosolic (c) and mitochondrial (m) LSU and SSU species are identified (note that the cLSU is split). B) 10% acrylamide gel as in A). Note that a mitochondrial 5S rRNA (m5S) is visualized in the M lane and, to a much lower extent, in the T lane. A band corresponding in size to cytosolic 5.8S rRNA is visible in an overloaded M lane; however, no cytosolic 5S (c5S) is detectable. (TIF) [file pone.0069532.s002.tif]

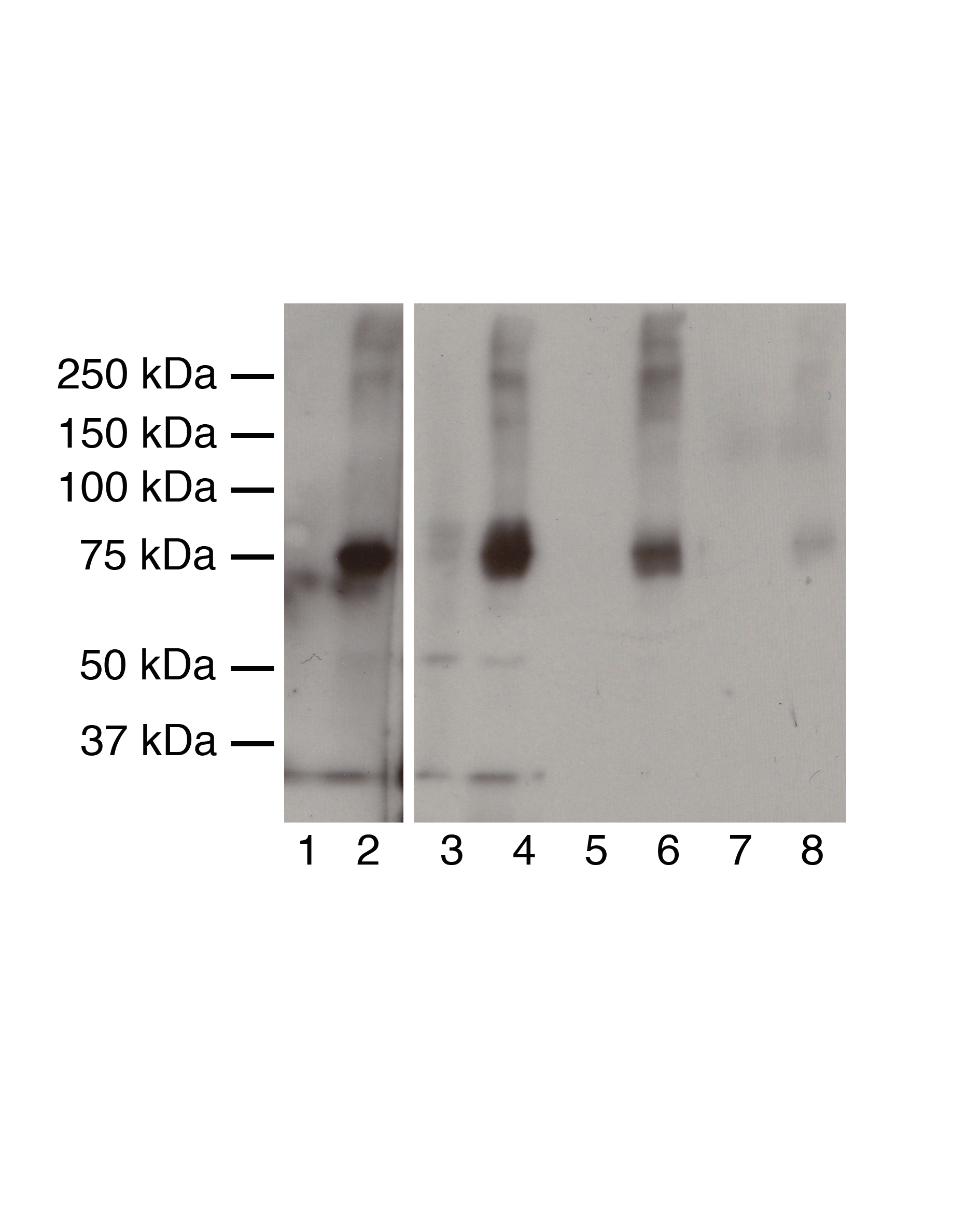

Supplement: Figure S2 — Western blot showing recognition of the recombinant [FeFe]-hydrogenase by the homologous anti-[FeFe]-hydrogenase antibody. Lanes 1, 3, 5 and 7: inclusion bodies from C41(DE) cells expressing empty pET-16b vector. Lanes 2, 4, 6 and 8: inclusion bodies from C41(DE) cells expressing recombinant A. castellanii [FeFe]-hydrogenase from pET-16b. Lanes 1 and 2: anti-His-tag antibody, exposed for a shorter period of time than lanes 3–8 in order to avoid overexposure. Lanes 3 and 4: anti [FeFe]-hydrogenase antibody only. Lanes 5 and 6: anti-[FeFe]-hydrogenase antibody incubated with inclusion bodies from C41(DE) cells expression empty pET-16b. Lanes 7 and 8: anti-[FeFe]-hydrogenase antibody incubated with inclusion bodies from C41(DE) cells expressing recombinant A. castellanii [FeFe]-hydrogenase from pET-16b. (TIF) [file pone.0069532.s003.tif]

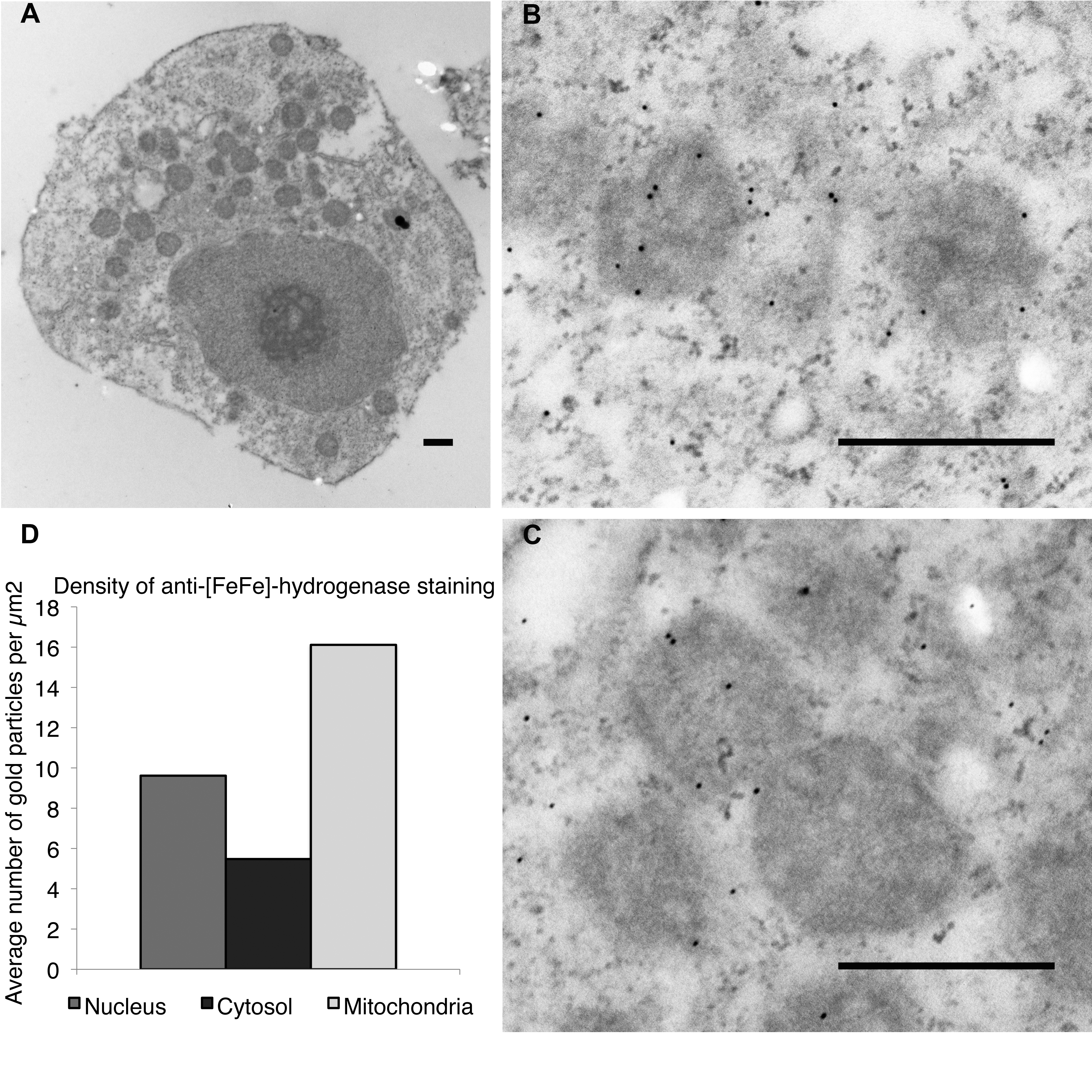

Supplement: Figure S3 — Immunogold localization of [FeFe]-hydrogenase in A. castellanii trophozoites exposed to anaerobic conditions for 24 hr. A. Whole cell fixed for immunogold staining; scale bar, 500 nm; this image has been cropped in order to show only the whole cell from which the insets shown were derived. B. and C. Magnified sections from the cell depicted in (A), showing gold particles corresponding to [FeFe]-hydrogenase localization; scale bar, 500 nm. D. Mean density of immunogold labeling in the cytosol, nucleus and mitochondria (8 cells). Brightness and contrast have been adjusted in each image to enhance visibility of the mitochondria and gold particles. (TIF) [file pone.0069532.s004.tif]

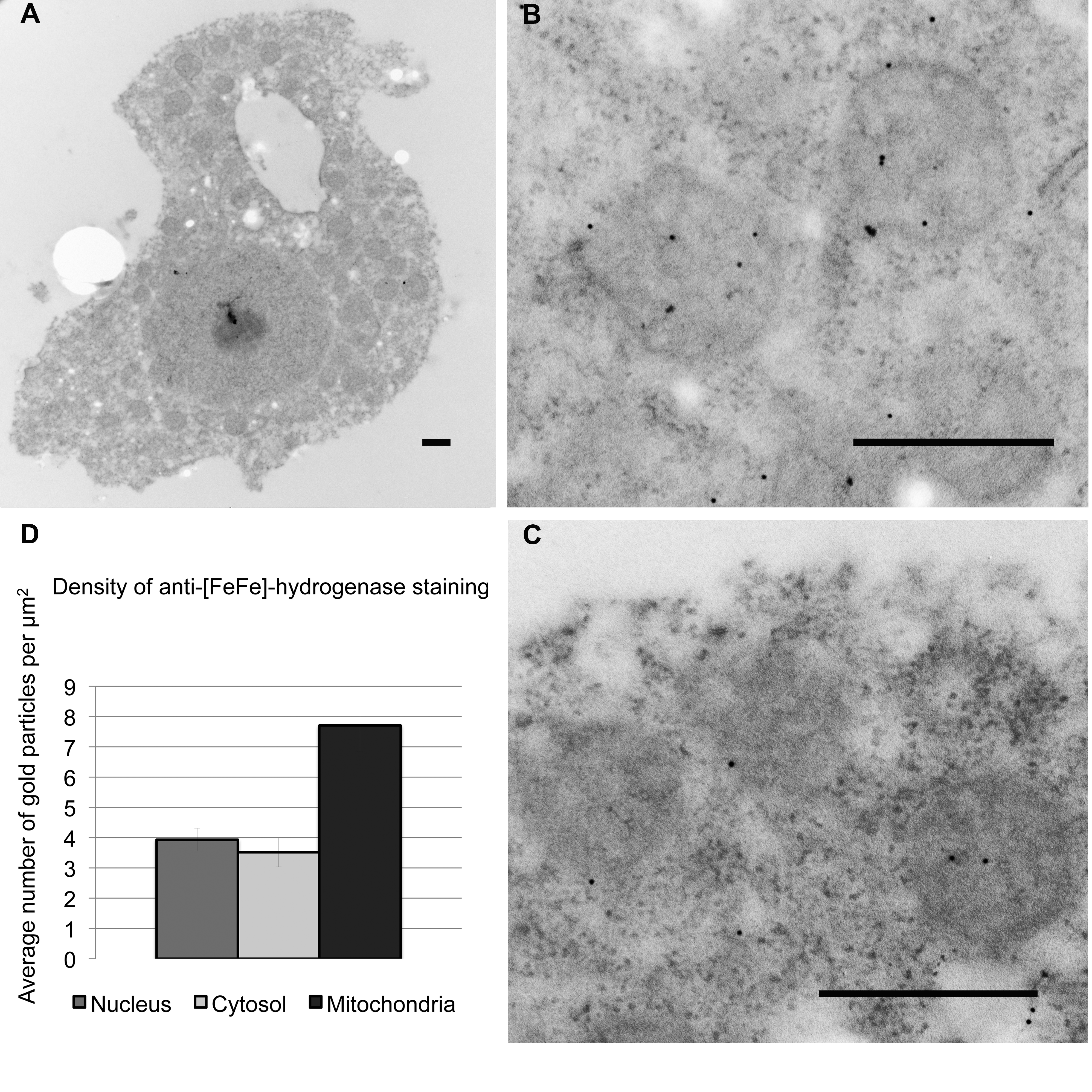

Supplement: Figure S4 — Immunogold localization of [FeFe]-hydrogenase in A. castellanii trophozoites exposed to anaerobic conditions for 6 hr. A. Whole cell fixed for immunogold staining; scale bar, 500 nm; this image has been cropped in order to show only the whole cell from which the insets shown were derived. B. and C. Magnified sections from the cell depicted in (A), showing gold particles corresponding to [FeFe]-hydrogenase localization; scale bar, 500 nm. D. Mean density of immunogold labeling in the cytosol, nucleus and mitochondria (7 cells). Brightness and contrast have been adjusted in each image to enhance visibility of the mitochondria and gold particles. (TIF) [file pone.0069532.s005.tif]

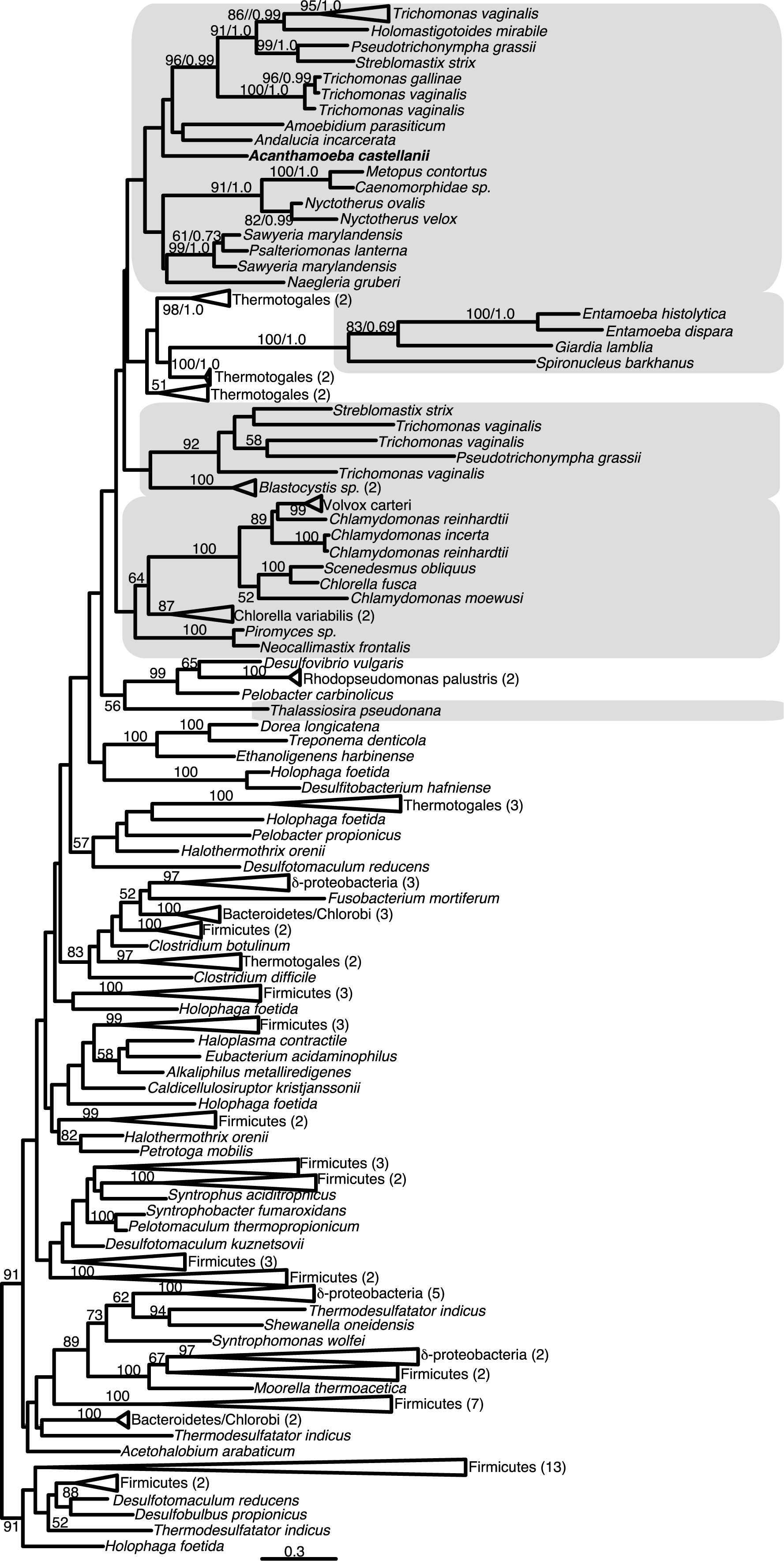

Supplement: Figure S5 — Phylogeny of [FeFe]-hydrogenase in eukaryotes and bacteria excluding long-branching taxa. Taxa forming a long-branching clade, corresponding to Clade B identified by Hug et al. (2010), have been excluded from these analyses. The topology shown is the ML tree generated by RAxML analyses; 328 sites were examined across 151 taxa. Bootstrap support values ≥50% and posterior probabilities ≥0.5 are shown. Eukaryotes are shaded gray. (TIF) [file pone.0069532.s006.tif]

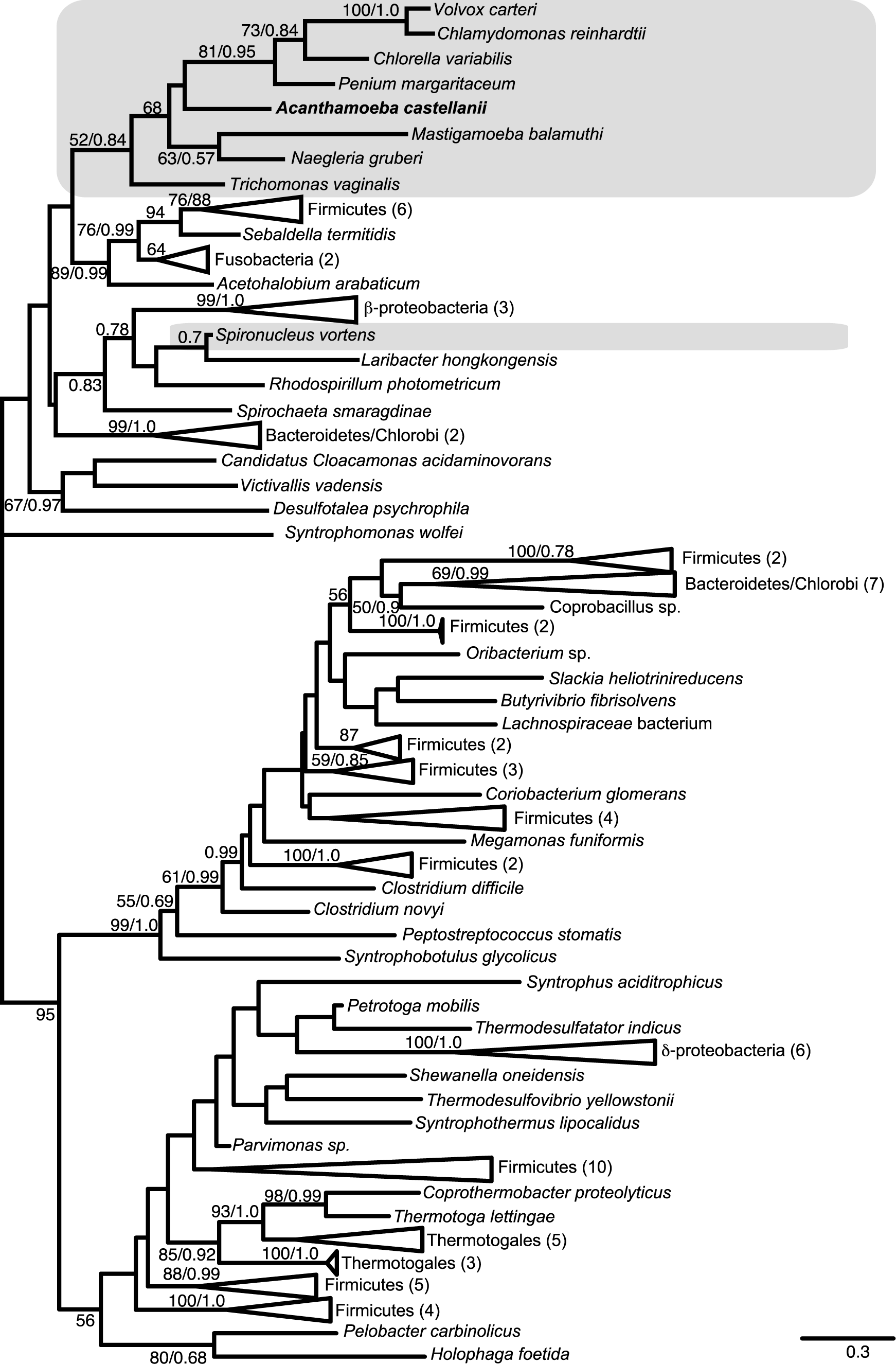

Supplement: Figure S6 — Phylogeny of HydE in eukaryotes and bacteria. The topology shown is the ML tree generated by RAxML analyses; 264 sites were examined across 109 taxa. Bootstrap support values ≥50% and posterior probabilities ≥0.5 are shown. Eukaryotes are shaded gray. (TIF) [file pone.0069532.s007.tif]

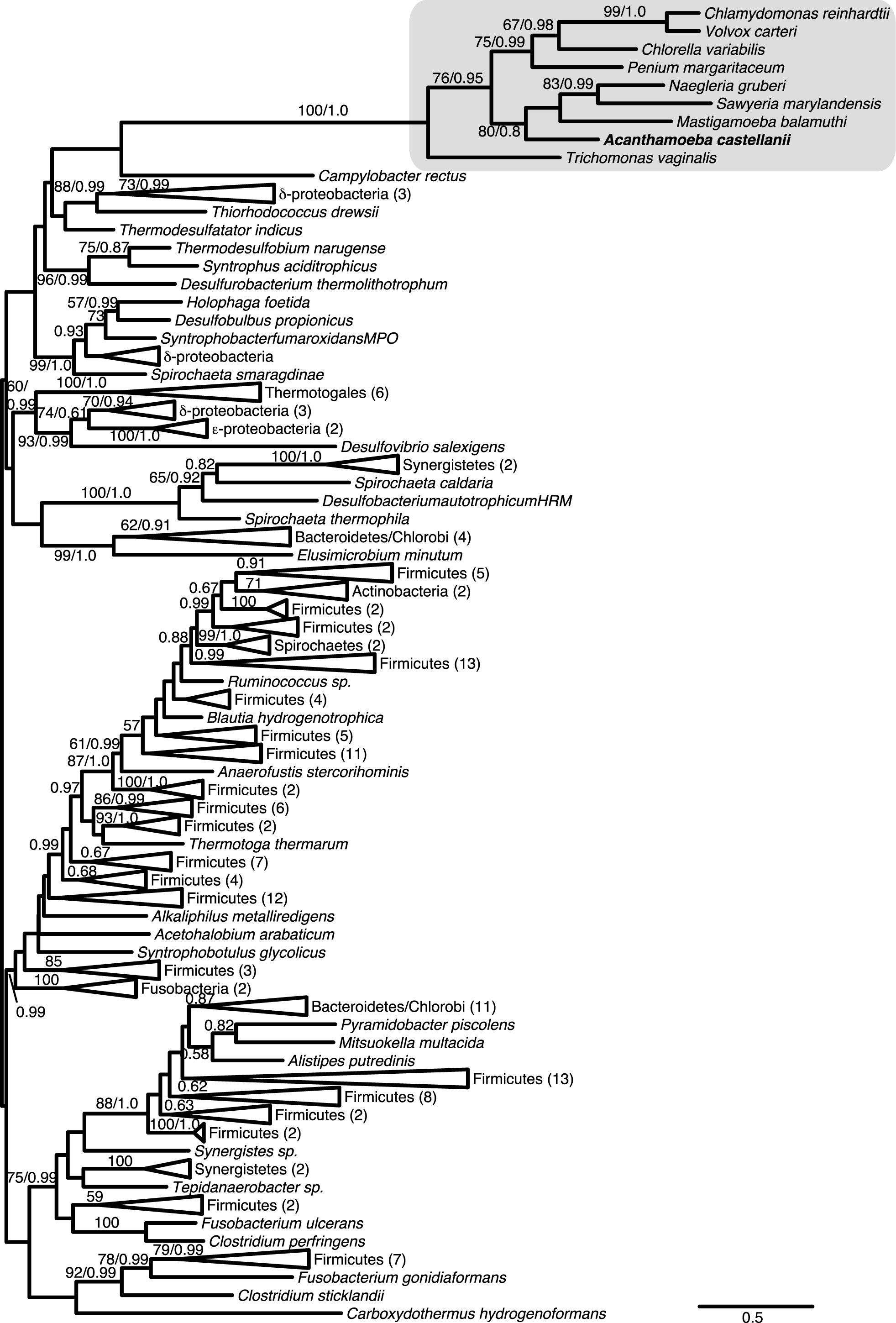

Supplement: Figure S7 — Phylogeny of HydF in eukaryotes and bacteria. The topology shown is the ML tree generated by RAxML analyses; 307 sites were examined across 196 taxa. Bootstrap support values ≥50% and posterior probabilities ≥0.5 are shown. Eukaryotes are shaded gray. (TIF) [file pone.0069532.s008.tif]

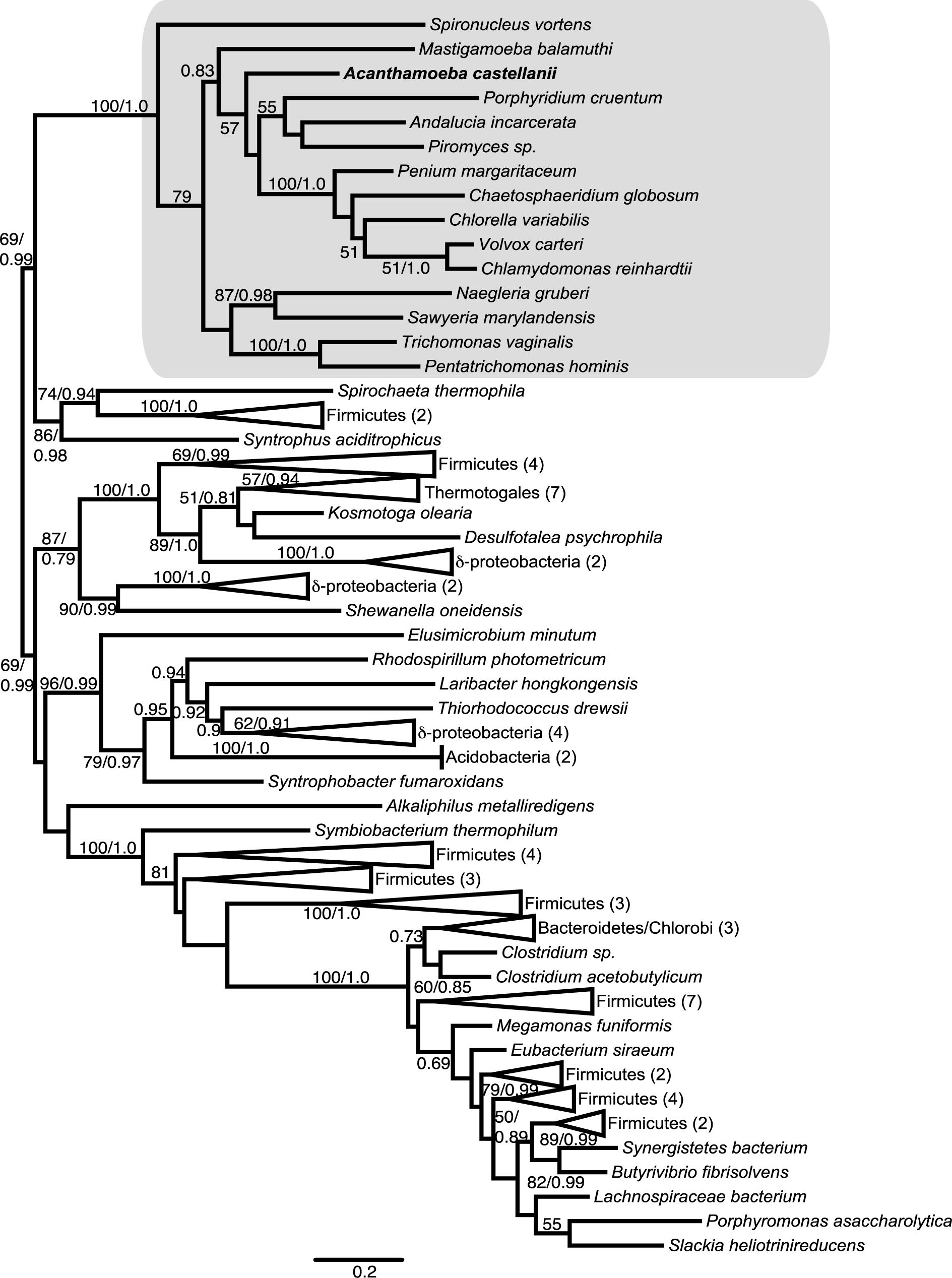

Supplement: Figure S8 — Phylogeny of HydG in eukaryotes and bacteria. The topology shown is the ML tree generated by RAxML analyses. 391 sites were examined across 87 taxa; bootstrap support values greater than or equal to 50%, and posterior probabilities greater than or equal to 0.5, are shown. Eukaryotes are shaded gray. (TIF) [file pone.0069532.s009.tif]

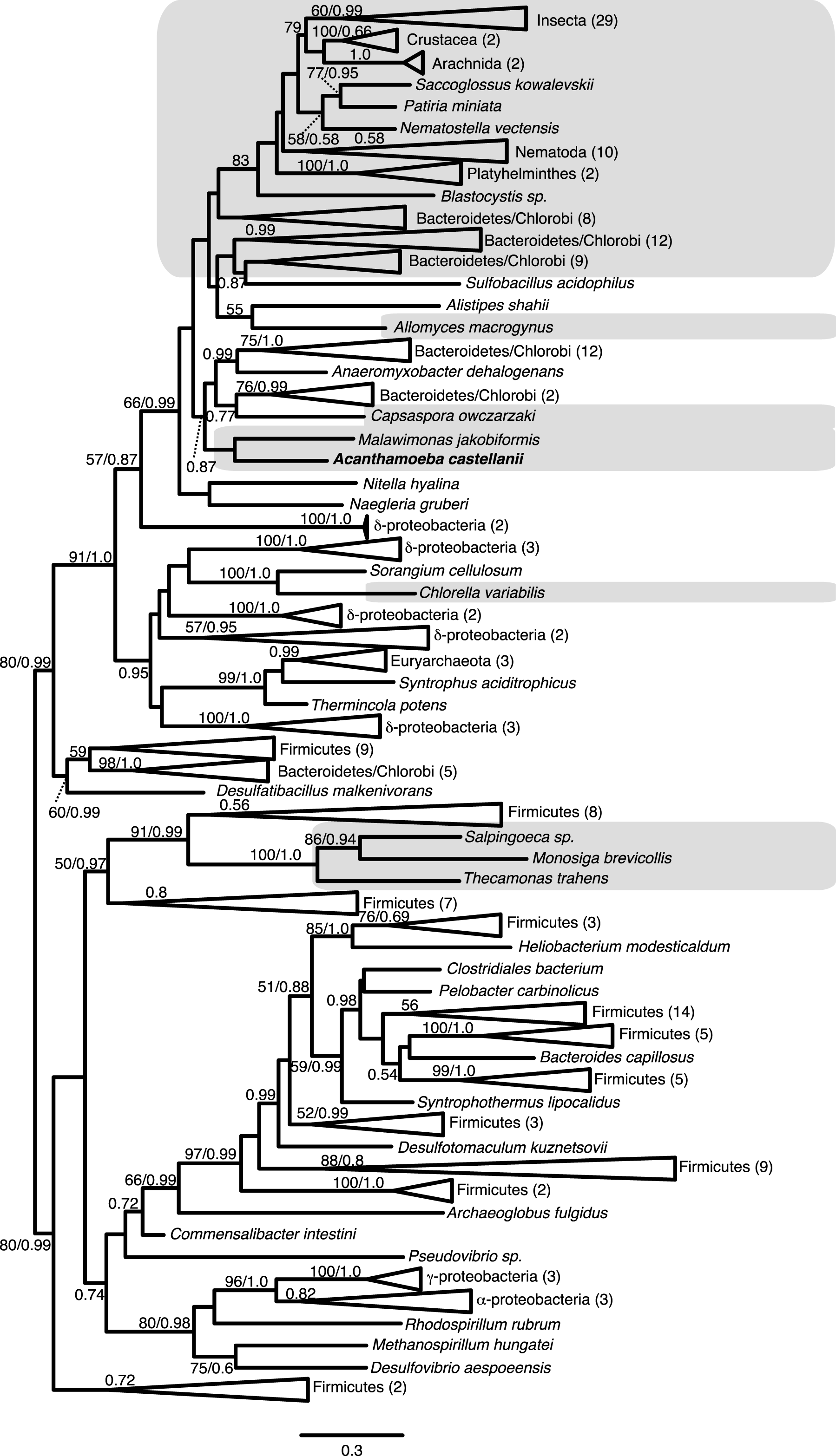

Supplement: Figure S9 — Phylogeny of ASCT1B in eukaryotes and bacteria. The topology shown is the ML tree generated by RAxML analyses; 315 sites were examined across 221 taxa. Bootstrap support values ≥50% and posterior probabilities ≥0.5 are shown. Eukaryotes are shaded gray. (TIF) [file pone.0069532.s010.tif]
