# Supplementary material for: Evidence for a Hydrogenosomal-Type Anaerobic ATP Generation Pathway in Acanthamoeba castellanii
Source: PLoS One. 2013 Sep 27;8(9):e69532. doi: 10.1371/journal.pone.0069532 (PMC3785491; doi:10.1371/journal.pone.0069532)
Supplement: Table S1 — Accession numbers of genes and ESTs encoding anaerobic energy generation enzymes. (DOCX) [file pone.0069532.s011.docx]

**Table S1.**

|  | Genomic DNA NCBI accession numbers | cDNA NCBI accession numbers |
| --- | --- | --- |
| [FeFe]-hydrogenase | AEYA01000219, (75972 – 75673, 75581 – 75413, 75351 – 75282, 75212 – 75041, 74960 – 74885, 74787 – 74630, 74547 – 74221, 74156 – 73995, 73905 – 73487, 73421 – 73279, 73195 – 73115) | EC110970, EC109037, GANQ00000000 |
| PFO | AEYA01001097 (15473 – 15542, 15611 – 15727, 15806 – 15924, 16047 – 16397, 16494 – 17192, 17297 – 17400, 17510 – 18330, 18435 – 18558, 19407 – 19554, 19630 – 20010) | EC107872, GANQ00000000 |
| HydE | AEYA01000219 (120492 – 120286, 120217 – 120163, 120101 – 119992, 119912 – 119849, 119787 – 119712, 119627 – 119387, 119326 – 119073, 118996 – 118803, 118730 – 118489) | EC100760, EC110418,  EC106931, GANQ00000000 |
| HydF | AEYA01000219 (120678 – 120792, 120860 – 120966, 120137 – 121153, 121228 – 121326, 121395 – 121532, 121601 – 121719, 121795 – 121907, 121970 – 122342, 122410 – 122701) | GANQ00000000 |
| HydG | AEYA01000219 (103720 – 103594, 103484 – 103336, 103238 – 102957, 102857 – 102737, 102658 – 101825, 101734 – 101583) | EC108023, EC108506, GANQ00000000 |
| ASCT 1A | AEYA01002158 (112023 – 111879, 111691 – 111528, 111428 – 111284. 111178 – 111086, 110997 – 110914, 110803 – 110631, 110497 – 110329, 110257 – 110153, 110030 – 109923, 109815 – 109735, 109566 – 109491, 109418 – 109332. 109220 – 109140, 109056 – 108922) | EC108981, EC106432, GANQ00000000 |
| ASCT 1B | AEYA01000777 (11122 – 11029, 10854 – 10782, 10599 – 10509, 10225 – 10043, 9865 – 9752, 9628 – 9510, 9356 – 9203, 9112 – 8934, 8822 – 8710, 8609 – 8497, 8335 – 8150) | EC101929, GANQ00000000 |
